# A Convenient Diels-Alder Approach toward Potential Polyketide-like Antibiotics Using α-Activated α,β-Unsaturated 4,4-Dimethyl-1-tetralones as Dienophiles

**DOI:** 10.3390/molecules28062739

**Published:** 2023-03-17

**Authors:** Chia-Jui Lee, Manickavasakam Ramasamy, Hsuan-Hao Kuan, Chien-Huang Wu, Chein-Chung Lee, Jinq-Chyi Lee, Kak-Shan Shia

**Affiliations:** Institute of Biotechnology and Pharmaceutical Research, National Health Research Institutes, Miaoli County 35053, Taiwan

**Keywords:** polyketides, MRSA, VRSA, dienophile, Diels–Alder cycloaddition, Lewis acid

## Abstract

Making use of a Diels–Alder approach based on various α,β-unsaturated 2-carbomethoxy-4,4-dimethyl-1-tetralones as novel dienophiles, the corresponding polycyclic adducts could be efficiently synthesized in good to high yields (74~99%) in the presence of Lewis acid (e.g., SnCl_4_). Accordingly, a synthetically useful platform is established to provide a focused aromatic polyketide-like library for screening of potential natural and non-natural antimicrobial agents.

## 1. Introduction

**Figure 1:**
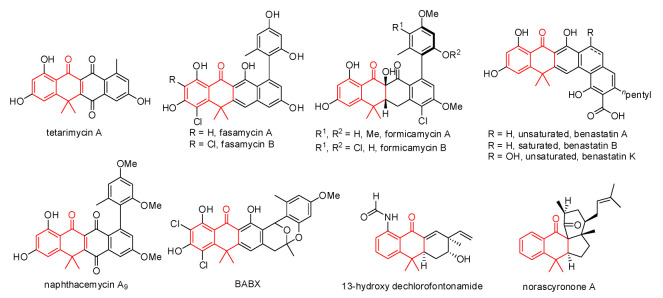
Aromatic polyketides containing a γ-*gem*-dimethyl-1-tetralone unit.

Naturally occurring polyketides isolated from Streptomyces species have an abundance of structural diversities and possess a variety of interesting bioactivities [1,2,3]. Among them, a family, featured with a 4,4-dimethyl-1-tetralone unit as highlighted in red in Figure 1, has been shown to have antimicrobial activities against Gram-positive pathogens, such as methicillin-resistant *Staphylococcus aureus* (*S. aureus)* (MRSA) and vancomycin-resistant *S. aureus* (VRSA) strains [4,5,6,7,8,9,10].

**Figure 2:**
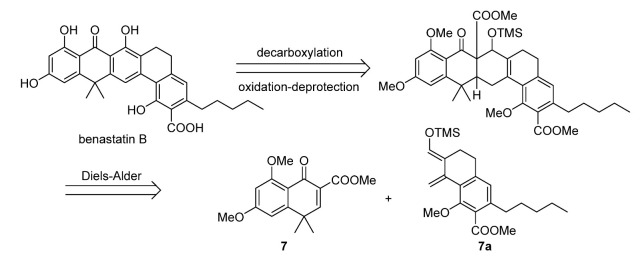
The retrosynthetic analysis of benastatin B through a plausible Diels–Alder approach.

Clinically, only a few antibiotics are able to cope with these resistant strains today. For example, daptomycin and linezolid are commonly used for the treatment of VRSA infection and vancomycin, teicolplanin, ceftaroline, and tigecycline are available for MRSA infection. It is highly conceivable that the prevalence of MRSA and VRSA strains may further evolve and develop resistance to current limited antibiotics, leading to the complete failure of clinical treatment in the future. Thus, developing more effective antibiotics to bolster an antimicrobial arsenal appears to be an urgent medical need for modern society. Along this axis, a plausible Diels–Alder approach to rapidly construct polycyclic core structures towards polyketide-like antibiotics was conceived. Using benastatin B as a typical example, retrosynthetic analysis (Figure 2) discloses that it can be disconnected into two major fragments, a dienophile **7** and created diene **7a**, by which the desired polycyclic skeleton can be simply established in one Diels–Alder operation to afford the corresponding adduct, of which decarboxylation and deprotection can be effected under acidic conditions, followed by Dess–Martin and/or DDQ oxidation to achieve the target molecule.

## 2. Results and Discussion

**Scheme 1:**
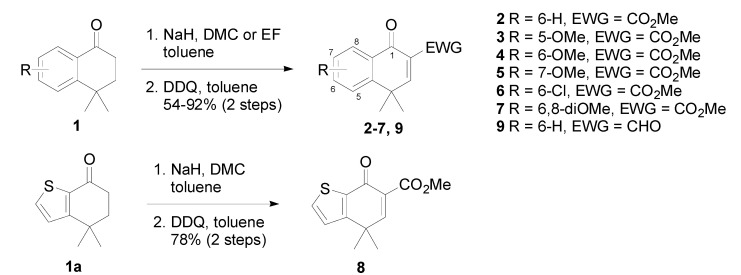
Preparation of α-activated dienophiles **2**–**9**.

Results of these studies are presented as follows. The project was initiated by the use of various 4,4-dimethyl-tetralones **1** or **1a** as starting material to provide dienophiles, which are either commercially available or readily prepared through several well-documented methodologies [4,5,6,11]. As typified by Scheme 1, using compounds **1** and **1a** as starting material, α-activated dienophiles **2**–**9** could be rapidly constructed based on a two-step synthetic sequence with good to high yields (54–92%), involving substitution with dimethyl carbonate (DMC) or ethyl formate (EF) in the presence of NaH, followed by 2,3-dichloro-5,6-dicyano-1,4-benzoquinone (DDQ) oxidation.

Inspired by our previous studies on Diels–Alder cycloaddition which can be easily promoted by the use of α-activated cross conjugated cycloalkenones as dienophiles [12,13,14,15,16,17,18,19,20] in the presence of various Lewis acids, a D–A model study based on dienophile **2** and 2,3-dimethyl-1,3-butadiene was then explored under catalysis with an array of Lewis acids, including SnCl_4_, BF_3_(OEt_2_), ZnCl_2_, TiCl_4_, ZnI_2_, and BCl_3_ (see Table S1 in SI). Among them, SnCl_4_ was found to be superior to others to facilitate the desired Diels–Alder reactions in terms of yields and milder reaction conditions. Further screening suggested that minor modifications with a raising reaction temperature from 0 °C to room temperature resulted in higher yields (Table 1, Entry 2, 91% vs. Entry 3, 98%). Thus, the reaction system (SnCl_4,_ ether, 0 °C to rt) indicated in Entry 3 was considered the method of choice and was adopted as a standard method for the current studies. Results and the scope of Diels–Alder reactions are compiled in Table 2 and discussed thereof as follows.

In general, we discovered that 4,4-dimethyl-tetral-1-ones **2**~**8** with an α-ester activating group could serve as dienophiles as efficient as their corresponding α-activated cross conjugated cycloalkenones with little influence by either an electron-donating or electron-withdrawing substituent(s) in the benzene ring in terms of yields, as seen in Entries 1 (98%), 3 (86%), and 5 (87%). However, the reaction rate for the presence of an electron-donating group(s) appeared to significantly retard the progress of reactions as compared with an electron-withdrawing group (Entry 3, 6-methoxy, 18 h vs. Entry 5, 6-Cl, 2 h). Apparently, these results might be attributed to the enhancement of the dienophilicity of dienophiles, thus lowering the energy of LUMO and accelerating reactions. However, it is hard to explain why the reaction rate of neutral dienophiles **2** and **8** (Entries 1, 7, 11, 14, and 17) was individually as efficient as the electron-deficient dienophile **6** (Entries 5, 10, 13, and 16), particularly when the *endo-to-ketone* transition state was considered a typically preferred mechanistic pathway due to a secondary orbital effect. Normally, addition of the 2-substituted dienes should result in more *para* than *anti-para* adducts as a result of the preferred electronic effects over the steric barrier in accordance with the *para* rule. Similar results were also observed in our cases, as seen in Entries 8 (**17**:**17a** = 74:26, 78%), 9 (**18**:**18a** = 69:31, 81%), and 10 (**19**:**19a** = 75:25, 79%), the ratio of which was tentatively determined based on the regiochemistry of *para* adduct **17**, whose structure was unambiguously identified by an X-ray crystallographic picture [21]. Since it was difficult to separate a pair of *para* and *anti-para* isomers, only the spectral data of major *para* adducts **17**, **18,** and **19** could be absolutely provided. *trans*-Piperylene was expected not to suffer any steric barrier and thus, the *endo-to-ketone* addition adducts **20** (Entry 11), **21** (Entry 12), and **22** (Entry 13), following the *ortho* rule, were exclusively obtained, the stereochemistry of which was respectively identified with a single-crystal X-ray analysis [22,23,24].

**Scheme 2:**
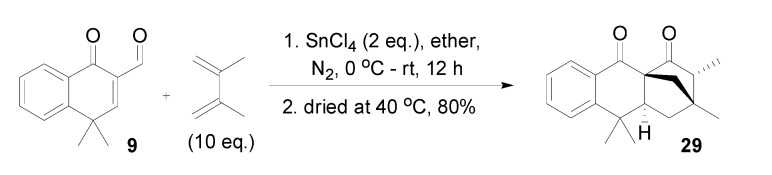
Diels–Alder reaction accompanied with unexpected rearrangements.

When a relatively more reactive Danishefsky’s diene was employed, ZnI_2_, a weaker Lewis acid, was elected instead to avoid polymerization, again furnishing the desired enones **27** and **28**, respectively, in good yields (63~72%) after acidic work-up conditions. The above results clearly demonstrate that this newly developed Diels–Alder approach in combination with an appropriate Lewis acid has great potential in synthesizing natural and non-natural polyketides containing a 4,4-dimethyl-1-tetralone unit. Meeting significant success in serving compound **2**~**8** as dienophiles, we further extended the methodology to dienophile **9** activated with an aldehyde group instead of an ester group [25]. Not unexpectedly, Diels–Alder reactions of **9** with various dienes tested in Table 2 under similar reaction conditions also gave rise to corresponding Diels–Alder adducts in moderate to good yields (63~90%, unpublished results). However, it was serendipitously found that when the reaction was complete (Scheme 2) and its reaction mixture was concentrated/dried directly in water bath at 40 °C instead of an usual work-up procedure, an unexpected product **29** was obtained exclusively in 80% yield, whose structure was unambiguously identified with an X-ray crystallographic analysis [26].

**Scheme 3:**
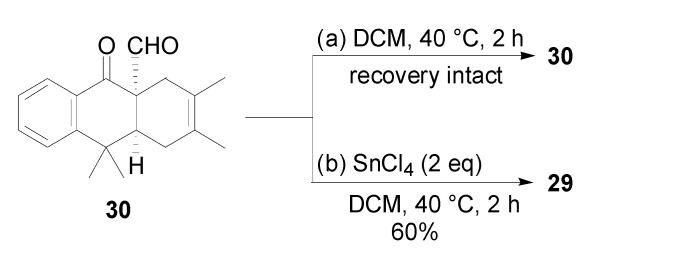
Diels–Alder adduct **30** was verified as an intermediate of **29**.

We speculated that **29** should be derived from the normal Diels–Alder adduct **30** during elevating temperature. Thus, compound **30**, obtained by a usual work-up as seen in Experimental 3.3., was subjected to two different reaction conditions (a) and (b), as seen in Scheme 3, to clarify which factor(s) really forced the reaction forward. Results clearly verified that compound **30** was smoothly converted to **29** in 60% under condition (b). Meanwhile, it was recovered intact under condition (a) in the absence of SnCl_4_.

**Scheme 4:**
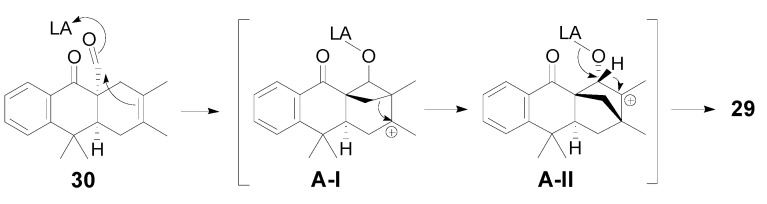
A proposed mechanism consisting of a domino D–A reaction and two 1,2-shifts.

Accordingly, the plausible mechanism is thus proposed as depicted in Scheme 4. The reaction sequence is believed to proceed with a consecutive Diels–Alder reaction and two 1,2-shift rearrangements. Upon the formation of Diels–Alder product **30**, its angular aldehyde moiety was complexed/catalyzed with LA (i.e., SnCl_4_) to form the oxonium ion, which in turn underwent an electrophilic addition with the alkene to give carbocation species A-I (like Prins reaction), by which a cascade of 1,2-hydride and 1,2-alkyl carbanion shifts sequentially occurred via species **A-II** to achieve diketone **29**, as similar to the previous report by Wang and coworkers [27].

## 3. Experimental Section

### 3.1. Materials and Reagents

All reactions were performed under N_2_ atmosphere unless otherwise stated. All reagents were employed as received without further purification. All solvents were dried and distilled by standard techniques. Diethyl ether was distilled from potassium and toluene was distilled from calcium hydride under N_2_. Analytical thin layer chromatography was performed on SiO_2_ 60 F-254 plates and flash column chromatography was carried out using SiO_2_ 60 (particle size 0.040–0.055 mm, 230–400 mesh). Visualization was performed under UV irradiation at 254 nm followed by staining with aqueous potassium permanganate and charring by heat gun. ^1^H and ^13^C-NMR spectra were recorded by Bruker Ascend 400 (400 MHz) or Bruker Ascend 600 (600 MHz). Chemical shifts were expressed in ppm using TMS in CDCl_3_ (*δ* = 0.00) with residual chlroform-d1 (*δ* = 7.26) as the internal standard in ^1^H-NMR spectra. ^13^C-NMR spectra were recorded in CDCl_3_, using the central resonances of CDCl_3_ (*δ* = 77.00) as the internal references. Multiplicities are recorded as s (singlet), d (doublet), t (triplet), q (quartet), quint (quintet), dd (doublet of doublets), dt (doublet of triplets), tt (triplet of triplets), ddd (doublet of doublet of doublets), m (multiplet), and br (broad). Coupling constants (*J*) were expressed in Hz. HRMS was obtained on a triple quadrupole mass analysis using electron ionization (EI) and electrospray ionization (ESI) source, and spectral data were recorded as m/z values. Copies of ^1^H and ^13^C-NMR spectra of the synthetic products and X-ray crystallographic data copies of the products (**17**, **20**, **21**, **22** and **29**) can be found in Supplementary Materials.

### 3.2. General Procedure for Preparation of Dienophiles ***2–9*** Using ***6*** as a Typical Example

A stirred solution of 6-chloro-4,4-dimethyl-1-tetralone (0.86 g, 4.12 mmol), NaH (0.51 g, 21.25 mmol), and dimehtyl carbonate (0.60 g, 6.67 mmol) in dry toluene (10 mL) was heated up to reflux. After the reaction was complete, sat. NH_4_Cl_(aq)_ was added to quench the reaction. The organic layer was separated and the aqueous layer was extracted with EA (2 × 20 mL). The organic portions were combined and dried over MgSO_4_, filtered and concentrated to give the residue, which was purified by chromatography on silical gel (*n*-hexane:ethyl acetate = 4:1) to afford 6-chloro-2-carbomethoxy-4,4-dimethyl-1-tertralone (0.88 g) in 80% yield, which was subsequently stirred in dry toluene (10 mL), followed by adding DDQ (0.75 g, 3.30 mmol) in one portion and kept at 80 °C under N_2_ for 16 h. After the reaction was complete, the reaction mixture was filtrated with celite and quenched with saturated NaHCO_3(aq)_ (5 mL) and extracted with EtOAc (3 × 10 mL). Organic layers were combined and washed with brine, dried over MgSO_4_, and then filtered and concentrated to give the crude residue, which was purified by chromatography on silical gel (*n*-hexane:ethyl acetate = 4:1) to afford dienophile **6** (0.54 g) in 62% yield.

#### 3.2.1. Methyl 6-Chloro-4,4-dimethyl-1-oxo-1,4-dihydronaphthalene-2-carboxylate (**6**)

^1^H NMR (CDCl_3_, 600 MHz): δ 8.14 (d, *J* = 8.4 Hz, 1H), 7.59 (s, 1H), 7.48 (d, *J* = 1.8 Hz, 1H), 7.36 (dd, *J* = 8.4, 1.8 Hz, 1H), 3.88 (s, 3H), 1.53 (s, 6H); ^13^C NMR (CDCl_3_, 150 MHz): δ 179.8, 165.3, 161.8, 149.8, 139.7, 129.3, 129.3, 129.1, 127.8, 126.2, 52.5, 37.6, 29.3; HRMS [ESI]^+^ calculated for C_14_H_13_ClNaO_3_: 287.0451 [M+Na]^+^; found: 287.0451.

#### 3.2.2. Methyl 5-Methoxy-4,4-dimethyl-1-oxo-1,4-dihydronaphthalene-2-carboxylate (**3**)

57% Yield; ^1^H NMR (CDCl_3_, 400 MHz): δ 7.89 (d, *J* = 8.0 Hz, 1H), 7.58 (s, 1H), 7.40 (t, *J* = 8.0 Hz, 1H), 7.12 (d, *J* = 8.0 Hz, 1H), 3.92 (s, 3H), 3.89 (s, 3H), 1.61 (s, 6H); ^13^C NMR (CDCl_3_, 150 MHz): δ 180.9, 165.7, 165.1, 157.4, 135.7, 132.4, 128.0, 127.4, 119.7, 115.3, 55.5, 52.4, 37.7, 24.9; HRMS [ESI]^+^ calculated for C_15_H_16_NaO_4_: 283.0946 [M+Na]^+^; found: 283.0950.

#### 3.2.3. Methyl 7-Methoxy-4,4-dimethyl-1-oxo-1,4-dihydronaphthalene-2-carboxylate (**5**)

74% Yield; ^1^H NMR (CDCl_3_, 600 MHz): δ 7.60 (d, *J* = 3.0 Hz, 1H), 7.57 (s, 1H), 7.38 (d, *J* = 8.4 Hz, 1H), 7.11 (dd, *J* = 8.4, 3.0 Hz, 1H), 3.83 (s, 3H), 3.80 (s, 3H), 1.45 (s, 6H); ^13^C NMR (CDCl_3_, 150 MHz): δ 180.7, 165.6, 162.4, 158.4, 141.0, 131.6, 129.2, 127.4, 121.7, 108.8, 55.5, 52.4, 37.2, 29.2; HRMS [ESI]^+^ calculated for C_15_H_16_NaO_4_: 283.0946 [M+Na]^+^; found: 283.0952.

#### 3.2.4. Methyl 4,4-Dimethyl-7-oxo-4,7-dihydrobenzo[b]thiophene-6-carboxylate (**8**)

77% Yield; ^1^H NMR (CDCl_3_, 600 MHz): δ 7.63 (d, *J* = 4.8 Hz, 1H), 7.50 (s, 1H), 7.09 (d, *J* = 4.8 Hz, 1H), 3.79 (s, 3H), 1.41 (s, 6H); ^13^C NMR (CDCl_3_, 150 MHz): δ 175.7, 165.3, 162.0, 156.5, 135.8, 134.4, 129.5, 125.8, 52.4, 38.8, 27.8; HRMS [ESI]^+^ calculated for C_12_H_12_NaO_3_S: 259.0405 [M+Na]^+^; found: 259.0402.

### 3.3. General Procedure for Preparation of Diels-Alder Adducts ***10–28*** Using ***10*** as a Typical Example

To a stirred solution of dienophile **2** (145 mg, 0.63 mmol) in dry ether (6 mL), 2,3-Dimethyl-1,3-butadiene (520 mg, 6.33 mmol) was added, followed by tin (IV) chloride (240 mg, 1.27 mmol) dropwise under N_2_ at 0 °C. The resulting mixture was warmed up to room temperature and stirred for another 2.5 h. After the reaction was complete, sat. NaHCO_3(aq)_ (1.0 mL) was added to quench the reaction. The organic layer was separated and the aqueous layer was extracted with EtOAc (3 × 10 mL). The organic portions were combined, washed with brine, dried over MgSO_4_, and then filtered and concentrated to give the crude residue, which was purified by chromatography on silical gel (*n*-hexane:ethyl acetate = 3:1) to afford adduct **10** (193 mg) in 98% yield.

#### 3.3.1. (4a*S*,9a*R*)-Methyl 2,3,9,9-Tetramethyl-10-oxo-1,4,4a,9,9a,10-hexahydroanthracene-4a-carboxylate (**10**)

98% yield; ^1^H NMR (CDCl_3_, 400 MHz): δ 8.06 (d, *J* = 8.0 Hz, 1H), 7.54 (m, 1H), 7.39 (d, *J* = 9.2 Hz, 1H), 7.30 (m, 1H), 3.64 (s, 3H), 2.76 (m, 1H), 2.69 (d, *J* = 16.8 Hz, 1H), 2.49 (d, *J* = 16.8 Hz, 1H), 2.17 (dd, *J* = 18.0, 6.8 Hz, 1H), 1.79 (dd, *J* = 18.0, 8.4 Hz, 1H), 1.64 (s, 3H), 1.52 (s, 3H), 1.36 (s, 3H), 1.34 (s, 3H); ^13^C NMR (CDCl_3_, 100 MHz): δ 195.2, 174.7, 149.8, 134.2, 130.0, 127.7, 126.8, 126.4, 123.4, 123.1, 57.4, 52.5, 44.8, 37.6, 36.8, 32.5, 32.3, 28.3, 18.7, 18.5; HRMS [ESI]^+^ calculated for C_20_H_24_O_3_: 312.1720; found: 312.1724.

#### 3.3.2. (4a*S*,9a*R*)-Methyl 8-Methoxy-2,3,9,9-tetramethyl-10-oxo-1,4,4a,9,9a,10-hexahydroanthracene-4a-carboxylate (**11**)

74% yield; ^1^H NMR (CDCl_3_, 600 MHz): δ 7.73 (d, *J* = 7.8 Hz, 1H), 7.27 (t, *J* = 7.8 Hz, 1H), 7.09 (d, *J* = 7.8 Hz, 1H), 3.84 (s, 3H), 3.62 (s, 3H), 2.67 (d, *J* = 16.8 Hz, 1H), 2.61 (dd, *J* = 9.0, 6.6 Hz, 1H), 2.47 (d, *J* = 16.8 Hz, 1H), 2.20 (m, 1H), 1.83 (m, 1H), 1.64 (s, 3H), 1.52 (s, 3H), 1.48 (s, 3H), 1.44 (s, 3H); ^13^C NMR (CDCl_3_, 150 MHz): δ 195.4, 174.7, 158.7, 136.6, 132.3, 127.2, 123.4, 123.0, 120.3, 117.1, 57.0, 55.5, 52.4, 46.8, 37.8, 36.8, 32.2, 27.9, 26.6, 18.7, 18.4; HRMS [ESI]^+^ calculated for C_21_H_26_NaO_4_: 365.1729 [M+Na]^+^; found: 365.1726.

#### 3.3.3. (4a*S*,9a*R*)-Methyl 7-Methoxy-2,3,9,9-Tetramethyl-10-Oxo-1,4,4a,9,9a,10-Hexahydroanthracene-4a-Carboxylate (**12**)

86% yield; ^1^H NMR (CDCl_3_, 400 MHz): δ 8.07 (d, *J* = 7.6 Hz, 1H), 6.85–6.82 (m, 2H), 3.86 (s, 3H), 3.65 (s, 3H), 2.76 (m, 1H), 2.71 (d, *J* = 16.8 Hz, 1H), 2.47 (d, *J* = 16.8 Hz, 1H), 2.15 (dd, *J* = 18.0, 6.8 Hz, 1H), 1.79 (dd, *J* = 18.0, 8.0 Hz, 1H), 1.63 (s, 3H), 1.51 (s, 3H), 1.34 (s, 3H), 1.33 (s, 3H); ^13^C NMR (CDCl_3_, 100 MHz): δ 193.9, 174.9, 164.3, 152.4, 130.6, 123.7, 123.4, 123.2, 112.0, 111.8, 57.1, 55.4, 52.5, 44.8, 37.9, 37.0, 32.5, 32.4, 28.2, 18.7, 18.5; HRMS [ESI]^+^ calculated for C_21_H_26_O_4_: 342.1826; found: 342.1826.

#### 3.3.4. (4a*S*,9a*R*)-Methyl 6-Methoxy-2,3,9,9-tetramethyl-10-oxo-1,4,4a,9,9a,10-hexahydroanthracene-4a-carboxylate (**13**)

75% yield; ^1^H NMR (CDCl_3_, 600 MHz): δ 7.54 (d, *J* = 3.0 Hz, 1H), 7.29 (d, *J* = 9.0 Hz, 1H), 7.12 (dd, *J* = 9.0, 3.0 Hz, 1H), 3.84 (s, 3H), 3.65 (s, 3H), 2.75 (dd, *J* = 7.8, 6.6 Hz, 1H), 2.68 (d, *J* = 16.8 Hz, 1H), 2.48 (d, *J* = 16.8 Hz, 1H), 2.16 (dd, *J* = 18.0, 6.6 Hz, 1H), 1.78 (dd, *J* = 18.0, 7.8 Hz, 1.64 (s, 3H), 1.52 (s, 3H), 1.32 (s, 6H); ^13^C NMR (CDCl_3_, 150 MHz): δ 195.3, 174.7, 157.9, 142.3, 130.9, 128.2, 123.5, 123.0, 122.7, 109.3, 57.5, 55.4, 52.6, 44.9, 37.1, 36.9, 32.5, 32.3, 28.3, 18.7, 18.5; HRMS [ESI]^+^ calculated for C_21_H_26_NaO_4_: 365.1729 [M+Na]^+^; found: 365.1732.

#### 3.3.5. (4a*S*,9a*R*)-Methyl 7-Chloro-2,3,9,9-tetramethyl-10-oxo-1,4,4a,9,9a,10-hexahydroanthracene-4a-carboxylate (**14**)

87% yield; ^1^H NMR (CDCl_3_, 400 MHz): δ 8.00 (d, *J* = 8.4 Hz, 1H), 7.34 (d, *J* = 1.2 Hz, 1H), 7.27 (dd, *J* = 8.4, 1.2 Hz, 1H), 3.64 (s, 3H), 2.75 (m, 1H), 2.70 (d, *J* = 16.0 Hz, 1H), 2.47 (d, *J* = 16.0 Hz, 1H), 2.18 (dd, *J* = 18.0, 6.8 Hz, 1H), 1.72 (dd, *J* = 18.0, 8.4 Hz, 1H), 1.63 (s, 3H), 1.51 (s, 3H), 1.36 (s, 3H), 1.33 (s, 3H); ^13^C NMR (CDCl_3_, 100 MHz): δ 194.0, 174.5, 151.5, 140.5, 129.5, 128.6, 127.0, 126.9, 123.3, 123.1, 57.2, 52.6, 44.9, 37.8, 36.9, 32.4, 32.3, 28.2, 18.6, 18.4; HRMS [ESI]^+^ calculated for C_20_H_23_ClO_3_: 346.1330; found: 346.1327.

#### 3.3.6. (4a*S*,9a*R*)-Methyl 5,7-Dimethoxy-2,3,9,9-tetramethyl-10-oxo-1,4,4a,9,9a,10-hexahydroanthracene-4a-carboxylate (**15**)

91% yield; ^1^H NMR (CDCl_3_, 400 MHz): δ 6.43 (d, *J* = 2.4 Hz, 1H), 6.34 (d, *J* = 2.4 Hz, 1H), 3.87 (s, 3H), 3.85 (s, 3H), 3.63 (s, 3H), 2.68 (dd, *J* = 8.4, 6.8 Hz, 1H), 2.66 (d, *J* = 16.4 Hz, 1H), 2.43 (d, *J* = 16.4 Hz, 1H), 2.12 (dd, *J* = 16.8, 6.8 Hz, 1H), 1.77 (m, 1H), 1.61 (s, 3H), 1.50 (s, 3H), 1.32 (s, 3H), 1.30 (s, 3H); ^13^C NMR (CDCl_3_, 100 MHz): δ 192.7, 175.1, 164.2, 162.7, 154.5, 123.6, 123.0, 144.1, 103.7, 96.3, 58.7, 56.0, 55.3, 52.4, 44.7, 38.4, 37.0, 33.1, 32.6, 28.8, 18.6, 18.4; HRMS [ESI]^+^ calculated for C_22_H_28_NaO_5_: 395.1834 [M+Na]^+^; found: 395.1835.

#### 3.3.7. (4a*R*,8a*S*)-Methyl 4,4,6,7-Tetramethyl-9-oxo-4,4a,5,8,8a,9-hexahydronaphtho[2,3-b]thiophene-8a-carboxylate (**16**)

83% yield; ^1^H NMR (CDCl_3_, 400 MHz): δ 7.65 (d, *J* = 5.2 Hz, 1H), 7.03 (d, *J* = 5.2 Hz, 1H), 3.69 (s, 3H), 2.85 (dd, *J* = 6.8, 4.8 Hz, 1H), 2.59 (d, *J* = 17.2 Hz, 1H), 2.43 (d, *J* = 17.2 Hz, 1H), 2.11 (m, 1H), 1.93 (m, 1H), 1.62 (s, 3H), 1.58 (s, 3H), 1.36 (s, 3H), 1.21 (s, 3H); ^13^C NMR (CDCl_3_, 100 MHz): δ 189.8, 173.6, 158.7, 135.3, 133.3, 126.8, 123.6, 123.2, 58.8, 52.6, 44.9, 37.7, 35.6, 31.2, 31.0, 26.4, 18.8, 18.5; HRMS [ESI]^+^ calculated for C_18_H_22_O_3_S: 318.1284; found: 318.1285.

#### 3.3.8. (4a*S*,9a*R*)-Methyl 2,9,9-Trimethyl-10-oxo-1,4,4a,9,9a,10-hexahydroanthracene-4a-carboxylate (**17**)

58% yield; ^1^H NMR (CDCl_3_, 400 MHz): δ 8.07 (d, *J* = 9.2 Hz, 1H), 7.55 (m, 1H), 7.40 (d, *J* = 8.0 Hz, 1H), 7.31 (m, 1H), 5.41 (m, 1H), 3.66 (s, 3H), 2.83 (m, 1H), 2.79 (m, 1H), 2.54 (d, *J* = 17.2 Hz, 1H), 2.17 (dd, *J* = 18.4, 6.8 Hz, 1H), 1.79 (dd, *J* = 18.4, 8.0 Hz, 1H), 1.58 (s, 3H), 1.38 (s, 3H), 1.37 (s, 3H); ^13^C NMR (CDCl_3_, 100 MHz): δ 195.3, 174.6, 149.8, 134.2, 132.0, 129.8, 127.8, 126.8, 126.5, 118.2, 56.6, 52.6, 44.4, 37.6, 32.5, 30.9, 30.6, 28.2, 23.3; HRMS [ESI]^+^ calculated for C_19_H_22_O_3_: 298.1563; found: 298.1564.

#### 3.3.9. (4a*S*,9a*R*)-Methyl 7-Methoxy-2,9,9-trimethyl-10-oxo-1,4,4a,9,9a,10-hexahydroanthracene-4a-carboxylate (**18**)

56% yield; ^1^H NMR (CDCl_3_, 600 MHz): δ 8.06 (d, *J* = 9.6 Hz, 1H), 6.83–6.80 (m, 2H), 5.39 (m, 1H), 3.84 (s, 3H), 3.64 (s, 3H), 2.83–2.73 (m, 2H), 2.51 (d, *J* = 17.4 Hz, 1H), 2.14 (dd, *J* = 18.6, 6.0 Hz, 1H), 1.77 (dd, *J* = 18.6, 8.4 Hz, 1H), 1.56 (s, 3H), 1.34 (s, 6H); ^13^C NMR (CDCl_3_, 150 MHz): δ 194.0, 174.8, 164.4, 152.4, 130.6, 123.5, 119.2, 118.4, 112.1, 111.8, 56.3, 55.4, 52.5, 44.3, 37.9, 32.3, 31.0, 30.7, 28.1, 23.3; HRMS [ESI]^+^ calculated for C_20_H_24_NaO_4_: 351.1572 [M+Na]^+^; found: 351.1571.

#### 3.3.10. (4a*S*,9a*R*)-Methyl 7-Chloro-2,9,9-trimethyl-10-oxo-1,4,4a,9,9a,10-hexahydroanthracene-4a-carboxylate (**19**)

59% yield; ^1^H NMR (CDCl_3_, 400 MHz): δ 7.92 (d, *J* = 8.0 Hz, 1H), 7.26 (d, *J* = 2.0 Hz, 1H), 7.19 (dd, *J* = 8.0, 2.0 Hz, 1H), 5.31 (m, 1H), 3.56 (s, 3H), 2.75–2.67 (m, 2H), 2.43 (d, *J* = 17.2 Hz, 1H), 2.08 (dd, *J* = 18.4, 6.8 Hz, 1H), 1.64 (dd, *J* = 18.4, 7.2 Hz, 1H), 1.48 (s, 3H), 1.29 (s, 3H), 1.26 (s, 3H); ^13^C NMR (CDCl_3_, 100 MHz): δ 194.1, 174.5, 151.5, 140.6, 131.9, 129.6, 128.4, 127.1, 127.0, 118.2, 56.4, 52.7, 44.6, 37.9, 32.3, 31.0, 30.7, 28.2, 23.2; HRMS [ESI]^+^ calculated for C_19_H_21_ClNaO_3_: 355.1077 [M+Na]^+^; found: 355.1077.

#### 3.3.11. (4*R*,4a*S*,9a*R*)-Methyl 4,9,9-Trimethyl-10-oxo-1,4,4a,9,9a,10-hexahydroanthracene-4a-carboxylate (**20**)

85% yield; ^1^H NMR (CDCl_3_, 400 MHz): δ 7.99 (d, *J* = 8.4 Hz, 1H), 7.49 (m, 1H), 7.31–7.28 (m, 2H), 5.63 (m, 1H), 5.47 (m, 1H), 3.62 (s, 3H), 2.85 (m, 1H), 2.70 (dd, *J* = 10.8, 6.8 Hz, 1H), 2.40 (m, 1H), 1.77 (m, 1H), 1.40 (s, 3H), 1.36 (d, *J* = 7.6 Hz, 3H), 1.33 (s, 3H); ^13^C NMR (CDCl_3_, 100 MHz): δ 195.1, 174.6, 147.8, 133.6, 132.0, 130.8, 127.4, 126.5, 126.4, 123.7, 59.9, 52.1, 49.3, 39.5, 38.6, 32.6, 29.0, 27.9, 17.2; HRMS [ESI]^+^ calculated for C_19_H_22_O_3_: 298.1563; found: 298.1569.

#### 3.3.12. (4*R*,4a*S*,9a*R*)-Methyl 7-Methoxy-4,9,9-trimethyl-10-oxo-1,4,4a,9,9a,10-hexahydroanthracene-4a-carboxylate (**21**)

87% yield; ^1^H NMR (CDCl_3_, 400 MHz): δ 8.01 (d, *J* = 8.4 Hz, 1H), 6.82 (dd, *J* = 8.4, 2.4 Hz, 1H), 6.74 (d, *J* = 2.4 Hz, 1H), 5.62 (m, 1H), 5.47 (m, 1H), 3.85 (s, 3H), 3.64 (s, 3H), 2.83 (m, 1H), 2.69 (dd, *J* = 10.8, 6.8 Hz, 1H), 2.39 (m, 1H), 1.78 (m, 1H), 1.39 (s, 3H), 1.37 (d, *J* = 7.6 Hz, 3H), 1.31 (s, 3H); ^13^C NMR (CDCl_3_, 100 MHz): δ 194.1, 174.8, 163.9, 150.5, 130.9, 130.1, 125.7, 123.9, 111.8, 111.6, 59.6, 55.3, 52.2, 49.3, 39.7, 38.9, 32.4, 28.9, 28.1, 17.4; HRMS [ESI]^+^ calculated for C_20_H_24_O_4_: 328.1669; found: 328.1672.

#### 3.3.13. (4*R*,4a*S*,9a*R*)-Methyl 7-Chloro-4,9,9-trimethyl-10-oxo-1,4,4a,9,9a,10-hexahydroanthracene-4a-carboxylate (**22**)

99% yield; ^1^H NMR (CDCl_3_, 400 MHz): δ 7.94 (d, *J* = 6.8 Hz, 1H), 7.28–7.25 (m, 2H), 5.62 (m, 1H), 5.47 (m, 1H), 3.64 (s, 3H), 2.85 (m, 1H), 2.70 (dd, *J* = 10.8, 6.8 Hz, 1H), 2.41 (m, 1H), 1.73 (m, 1H), 1.40 (s, 3H), 1.35 (d, *J* = 7.6 Hz, 3H), 1.32 (s, 3H); ^13^C NMR (CDCl_3_, 100 MHz): δ 194.1, 174.4, 149.7, 139.9, 130.8, 130.5, 129.1, 127.1, 126.6, 123.5, 59.8, 52.3, 49.1, 39.5, 38.8, 32.4, 28.9, 27.9, 17.1; HRMS [ESI]^+^ calculated for C_19_H_21_ClO_3_: 332.1174; found: 332.1176.

#### 3.3.14. (5a*S*,11a*R*)-Methyl 11,11-Dimethyl-6-oxo-1,4,5,5a,6,11,11a,12-octahydrotetracene-5a-carboxylate (**23**)

94% yield; ^1^H NMR (CDCl_3_, 400 MHz): δ 8.07 (d, *J* = 8.0 Hz, 1H), 7.55 (m, 1H), 7.39 (d, *J* = 6.8 Hz, 1H), 7.30 (m, 1H), 5.69–5.61 (m, 2H), 3.65 (s, 3H), 2.85 (m, 1H), 2.68–2.59 (m, 3H), 2.49–2.42 (m, 3H), 2.13 (dd, *J* = 18.0, 6.8 Hz, 1H), 1.75 (dd, *J* = 18.0, 8.4 Hz, 1H), 1.38 (s, 3H), 1.36 (s, 3H); ^13^C NMR (CDCl_3_, 100 MHz): δ 194.9, 174.5, 149.7, 134.2, 129.9, 127.7, 126.8, 126.5, 124.2, 124.0, 123.4, 123.2, 57.2, 52.5, 44.6, 37.6, 35.0, 32.5, 30.9, 30.6, 30.4, 28.3; HRMS [ESI]^+^ calculated for C_22_H_24_O_3_: 336.1720; found: 336.1720.

#### 3.3.15. (5a*S*,11a*R*)-Methyl 9-Methoxy-11,11-dimethyl-6-oxo-1,4,5,5a,6,11,11a,12-octahydrotetracene-5a-carboxylate (**24**)

71% yield; ^1^H NMR (CDCl_3_, 400 MHz): δ 8.07 (d, *J* = 6.4 Hz, 1H), 6.84–6.81 (m, 2H), 5.68–5.60 (m, 2H), 3.86 (s, 3H), 3.65 (s, 3H), 2.84 (m, 1H), 2.65 (d, *J* = 15.6 Hz, 1H), 2.56 (m, 2H), 2.46–2.42 (m, 3H), 2.12 (dd, *J* = 18.0, 6.8 Hz, 1H), 1.74 (dd, *J* = 18.0, 8.0 Hz, 1H), 1.36 (s, 3H), 1.35 (s, 3H); ^13^C NMR (CDCl_3_, 100 MHz): δ 193.6, 174.7, 164.4, 152.4, 130.6, 124.3, 124.0, 123.7, 123.4, 123.3, 112.0, 111.8, 57.0, 55.4, 52.5, 44.6, 37.9, 35.2, 32.4, 30.9, 30.6, 30.6, 28.2; HRMS [ESI]^+^ calculated for C_23_H_26_O_4_: 366.1826; found: 366.1822.

#### 3.3.16. (5a*S*,11a*R*)-Methyl 9-Chloro-11,11-dimethyl-6-oxo-1,4,5,5a,6,11,11a,12-octahydrotetracene-5a-carboxylate (**25**)

76% yield; ^1^H NMR (CDCl_3_, 400 MHz): δ 8.01 (d, *J* = 8.4 Hz, 1H), 7.35 (d, *J* = 2.0 Hz, 1H), 7.28 (dd, *J* = 8.4, 2.0 Hz, 1H), 5.69–5.60 (m, 2H), 3.65 (s, 3H), 2.83 (dd, *J* = 8.8, 6.8 Hz, 1H), 2.68 (d, *J* = 16.0 Hz, 1H), 2.58–2.52 (m, 2H), 2.47–2.39 (m, 3H), 2.13 (dd, *J* = 18.0, 6.8 Hz, 1H), 1.69 (dd, *J* = 18.0, 8.8 Hz, 1H), 1.38 (s, 3H), 1.35 (s, 3H); ^13^C NMR (CDCl_3_, 100 MHz): δ 193.8, 174.4, 151.4, 140.6, 129.6, 128.6, 127.1, 127.0, 124.2, 124.0, 123.2, 123.2, 57.1, 52.6, 44.8, 37.9, 35.1, 32.3, 30.8, 30.6, 30.5, 28.3; HRMS [ESI]^+^ calculated for C_22_H_23_ClO_3_: 370.1330; found: 370.1335.

#### 3.3.17. (4a*R*,10a*S*)-Methyl 4,4-Dimethyl-11-oxo-4,4a,5,6,9,10,10a,11-octahydroanthra[2,3-b]thiophene-10a-carboxylate (**26**)

99% yield; ^1^H NMR (CDCl_3_, 400 MHz): δ 7.66 (d, *J* = 5.2 Hz, 1H), 7.05 (d, *J* = 5.2 Hz, 1H), 5.69–5.63 (m, 2H), 3.71 (s, 3H), 2.93 (dd, *J* = 6.8, 4.8 Hz, 1H), 2.58–2.39 (m, 6H), 2.10 (m, 1H), 1.87 (m, 1H), 1.38 (s, 3H), 1.24 (s, 3H); ^13^C NMR (CDCl_3_, 100 MHz): δ 189.6, 173.5, 158.7, 135.4, 133.3, 126.9, 124.3, 124.0, 123.6, 123.3, 58.7, 52.7, 44.8, 37.8, 33.9, 31.0, 31.0, 30.7, 29.4, 26.5; HRMS [ESI]^+^ calculated for C_20_H_22_O_3_S: 342.1284; found: 342.1285.

#### 3.3.18. (4a*S*,9a*R*)-Methyl 9,9-Dimethyl-2,10-dioxo-1,2,4a,9,9a,10-hexahydroanthracene-4a-carboxylate (**27**)

63% yield; ^1^H NMR (CDCl_3_, 400 MHz): δ 8.06 (d, *J* = 10.4 Hz, 1H), 7.64 (m, 1H), 7.44–7.40 (m, 2H), 7.14 (d, *J* = 13.6 Hz, 1H), 6.23 (d, *J* = 13.6 Hz, 1H), 3.76 (s, 3H), 3.23 (dd, *J* = 12.4, 6.8 Hz, 1H), 2.71 (dd, *J* = 22.8, 6.8 Hz, 1H), 2.44 (dd, *J* = 22.8, 12.4 Hz, 1H), 1.43 (s, 3H), 1.30 (s, 3H); ^13^C NMR (CDCl_3_, 100 MHz): δ 197.0, 192.6, 171.5, 150.0, 148.0, 135.2, 130.2, 129.8, 128.5, 127.1, 125.6, 59.1, 53.7, 47.4, 37.3, 37.3, 29.8, 28.9; HRMS [ESI]^+^ calculated for C_18_H_18_NaO_4_: 321.1103 [M+Na]^+^; found: 321.1103.

#### 3.3.19. (4a*R*,8a*S*)-Methyl 4,4-Dimethyl-6,9-dioxo-4,4a,5,6,8a,9-hexahydronaphtho[2,3-b]thiophene-8a-carboxylate (**28**)

72% yield; ^1^H NMR (CDCl_3_, 400 MHz): δ 7.77 (d, *J* = 5.2 Hz, 1H), 7.07 (d, *J* = 5.2 Hz, 1H), 7.02 (d, *J* = 10.0 Hz, 1H), 6.25 (d, *J* = 10.0 Hz, 1H), 3.79 (s, 3H), 3.27 (m, 1H), 2.67–2.53 (m, 2H), 1.43 (s, 3H), 1.26 (s, 3H); ^13^C NMR (CDCl_3_, 100 MHz): δ 196.8, 185.6, 171.5, 159.9, 146.9, 137.3, 133.7, 130.6, 126.6, 60.1, 53.7, 48.6, 37.8, 37.2, 29.3, 27.7; HRMS [ESI]^+^ calculated for C_16_H_16_NaO_4_S: 327.0667 [M+Na]^+^; found: 327.0665.

#### 3.3.20. (2*R*,4a*S*,9a*S*)-2,3,9,9-Tetramethyl -2,3,9,9a-tetrahydro-1H-2,4a-methanoanthracene-4,10-dione (**29**)

80% yield; ^1^H NMR (CDCl_3_, 400 MHz): δ 7.95 (d, *J* = 7.6 Hz, 1H), 7.51 (m, 1H), 7.37–7.30 (m, 2H), 2.18–2.05 (m, 4H), 1.93 (m, 1H), 1.59 (m, 1H), 1.35 (s, 3H), 1.27 (s, 3H), 1.11 (s, 3H), 1.06 (d, *J* = 7.6 Hz, 3H); ^13^C NMR (CDCl_3_, 100 MHz): δ 213.6, 198.2, 151.9, 133.8, 133.7, 127.6, 126.8, 123.7, 68.9, 53.1, 46.2, 46.1, 44.6, 37.4, 32.6, 28.2, 24.9, 19.4, 9.9; HRMS [ESI]^+^ calculated for C_19_H_22_NaO_2_: 305.1517 [M+Na]^+^; found: 305.1521.

## 4. Conclusions

In this work, we have demonstrated that a newly synthetic method through Diels–Alder addition to afford highly stereoselective and functionalized adducts has been developed, by which a library of potential natural and/or non-natural antibiotics, containing a 4,4-dimethyl-1-tetralone unit, can be rapidly built up for screening initial hit compounds for further peripherally structural modifications in the hope that advanced compounds with potent antimicrobial activities, particularly against Gram-positive pathogens, can be discovered.

## Figures and Tables

**Table 1:** Screening of Diels–Alder cycloaddition conditions *^a^*.

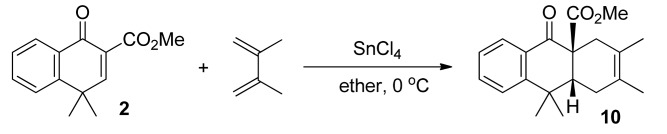				
Entry	Diene (equiv.)	SnCl_4_ (equiv.)	Time (h)	Yield (%) ^*b*^
1	20	2.5	3	84
2	10	2	5	91
3 ^*c*^	10	2	2.5	98
4 ^*c*^	5	2	3	93
5 ^*c*^	2	2	5	71

*^a^* Reactions were performed using dienophile **2**, 2,3-dimethyl-1,3-butadiene and SnCl_4_ in ether at 0 °C under N_2_ atmosphere. *^b^* Isolated yields. *^c^* Reactions were initiated at 0 °C and then gradually warmed up to rt.

**Table 2:** Scope of dienophiles and dienes applied for Diels–Alder cycloaddition *^a^*.

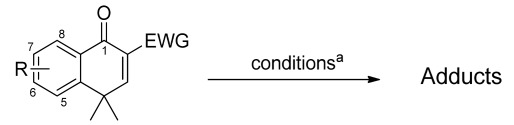					
Entry	Dienophile	Diene	t (h)	Product	Yield (%) *^b^*
1	2	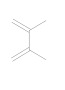	2.5	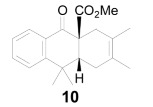	98
2	3	,,	29	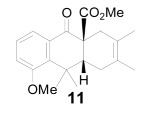	74 *^c^*
3	4	,,	18	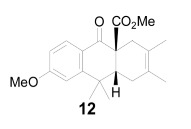	86
4	5	,,	18	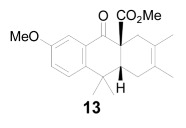	75
5	6	,,	2	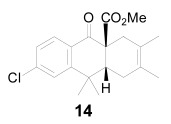	87
6	7	,,	24	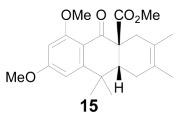	91
7	8	,,	2	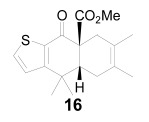	83
8	2	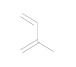	2	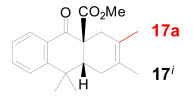	78 *^d^*
9	4	,,	16	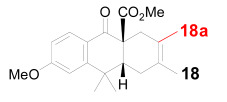	81 *^e^*
10	6	,,	1.5	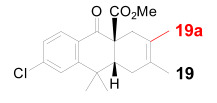	79 *^f^*
11	2	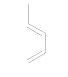	2	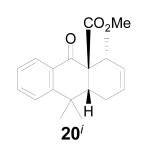	85
12	4	,,	16	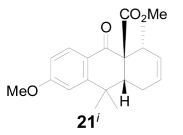	87
13	6	,,	1.5	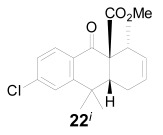	99
14	2	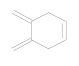	2	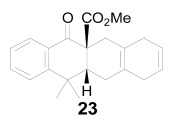	94
15	4	,,	18	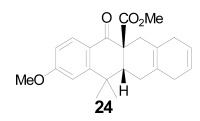	71
16	6	,,	2.5	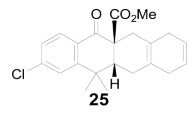	76
17	8	,,	2.5	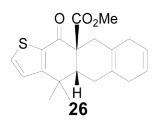	99
18	2	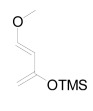	15	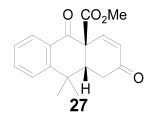	63 *^g,h^*
19	8	,,	18	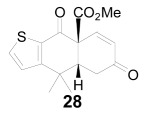	72 *^g,h^*

*^a^* All reactions were performed using dienophile (0.42~0.63 mmol), diene (10 eq), and SnCl_4_ (2 eq) in ether (6 mL) at 0 °C and then warmed up to room temperature under N_2_ atmosphere. *^b^* Isolated yields. *^c^* 20 equiv. of diene was used. *^d^* The ratio of *para* **17** and *anti-para* **17a** was 74/26. *^e^* The ratio of *para* **18** and *anti-para* **18a** was 69/31. *^f^* The ratio of *para* **19** and *anti-para* **19a** was 75/25. *^g^* ZnI_2_ was used instead. *^h^* 2N HCl (aq.) was required for further hydrolysis. *^i^* The relative configuration was unambiguously determined by a single-crystal X-ray analysis.

## Data Availability

The data present in this study are available on request from the corresponding authors.

## Supplementary Materials

The following supporting information can be downloaded at: https://www.mdpi.com/article/10.3390/molecules28062739/s1, Copies of ^1^H and ^13^C NMR spectra of the synthetic products **3**, **5**, **6**, **8** and **10**–**29** as well as copies of the X-ray crystallographic structure of synthetic products **17**, **20**–**22,** and **29** are available online. Table S1: Screening of Diels-Alder cycloaddition conditions.
