# Supplementary material for: A Convenient Diels-Alder Approach toward Potential Polyketide-like Antibiotics Using α-Activated α,β-Unsaturated 4,4-Dimethyl-1-tetralones as Dienophiles
Source: Molecules. 2023 Mar 17;28(6):2739. doi: 10.3390/molecules28062739 (PMC10057133; doi:10.3390/molecules28062739)
Supplement: Supplementary file 1 [file molecules-28-02739-s001.zip › molecules-2243116-supplementary.pdf]

Supporting Inofmation

A Convenient Diels-Alder Approach Toward Potential Polyketide-like Antibiotics Using α-Activated α,β-Unsaturated 4,4-Dimethyl-1-tetralones as Dienophiles

Chia-Jui Lee, Manickavasakam Ramasamy, Hsuan-Hao Kuan, Chien-Huang Wu, Chein-Chung Lee, Jinq-Chyi Lee* and Kak-Shan Shia*

E-mail: ksshia@nhri.edu.tw; jinqchyi@nhri.edu.tw

Contents

^{1}H and ^{13}C NMR spectra of compounds **3,5,6,8**-------------------------------------S1-S4

^{1}H and ^{13}C NMR spectra of compounds **10–29**------------------------------------S5-S24

X-ray crystallographic analysis of compounds **17,20-22,29**---------------------S25-S27

Table S1. Screening of Diels-Alder cycloaddition with different Lewis acids --S28

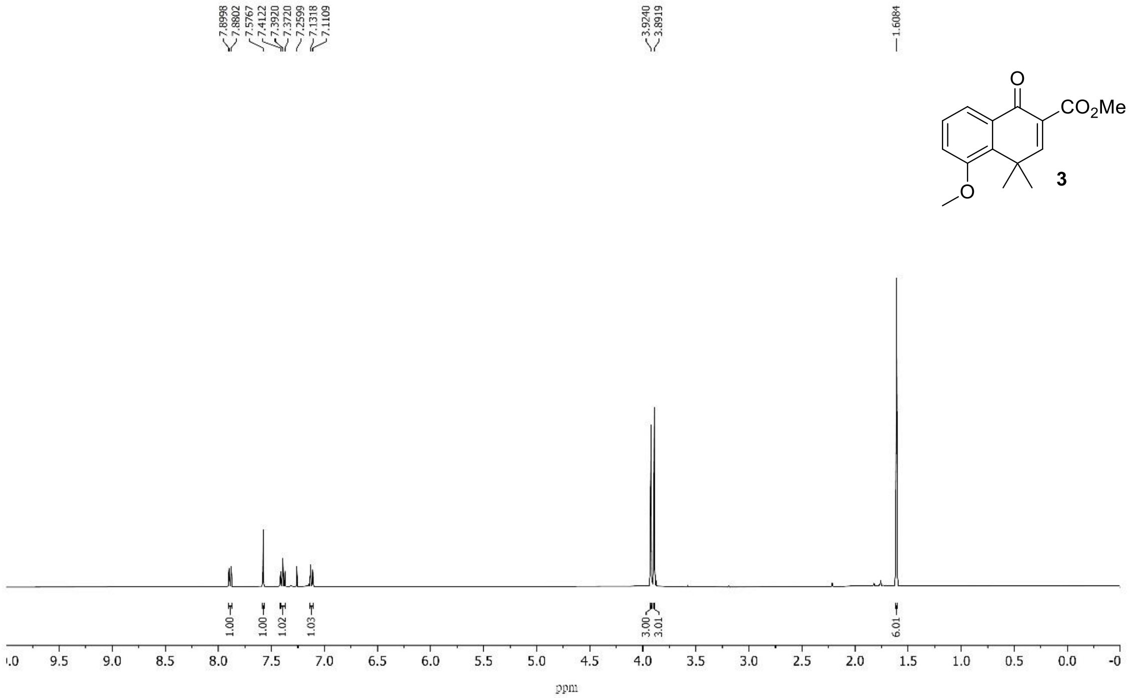

^1H NMR of compound **3**

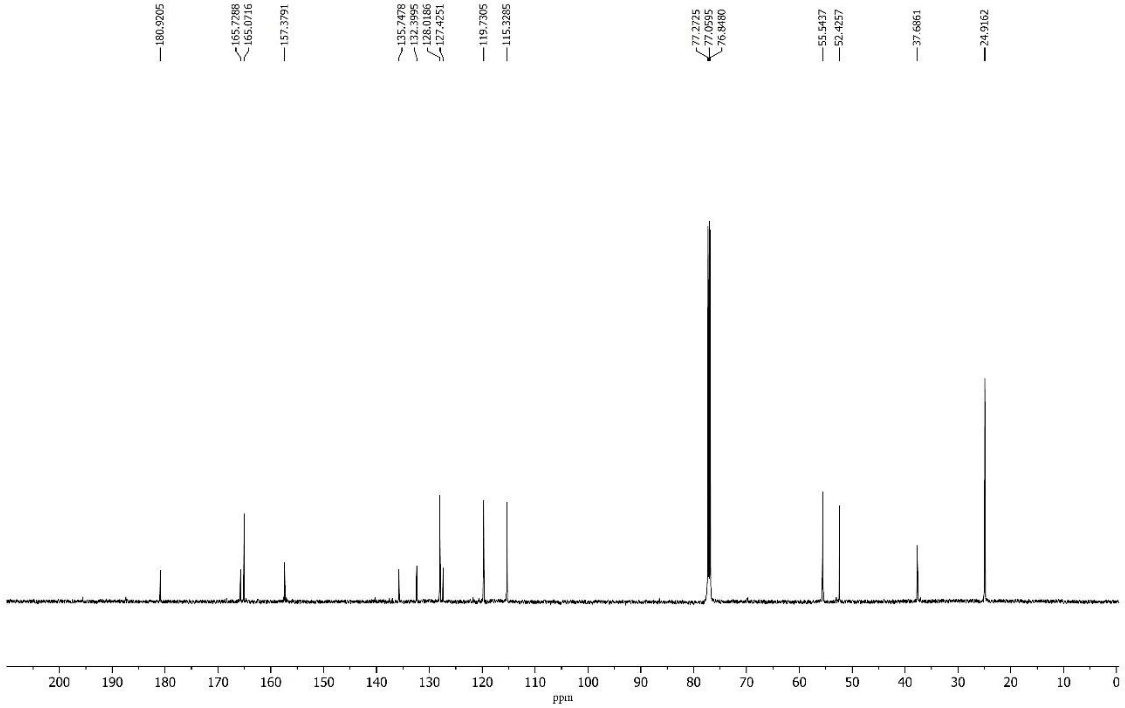

^{13}C NMR of compound **3**

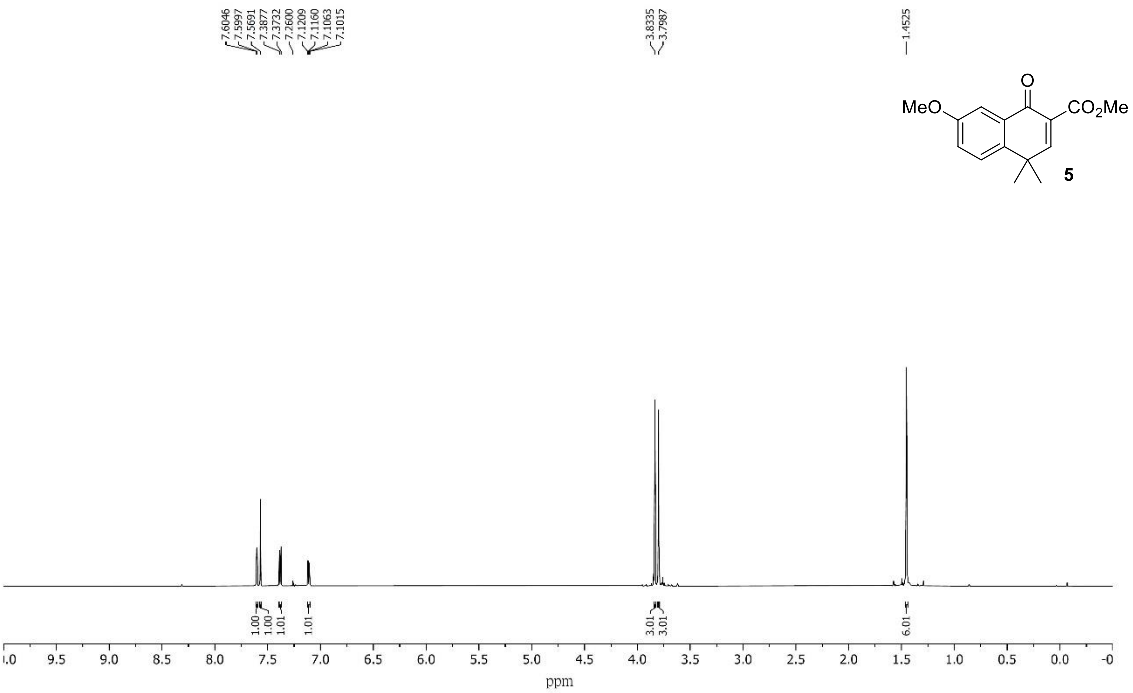

^{1}H NMR of compound **5**

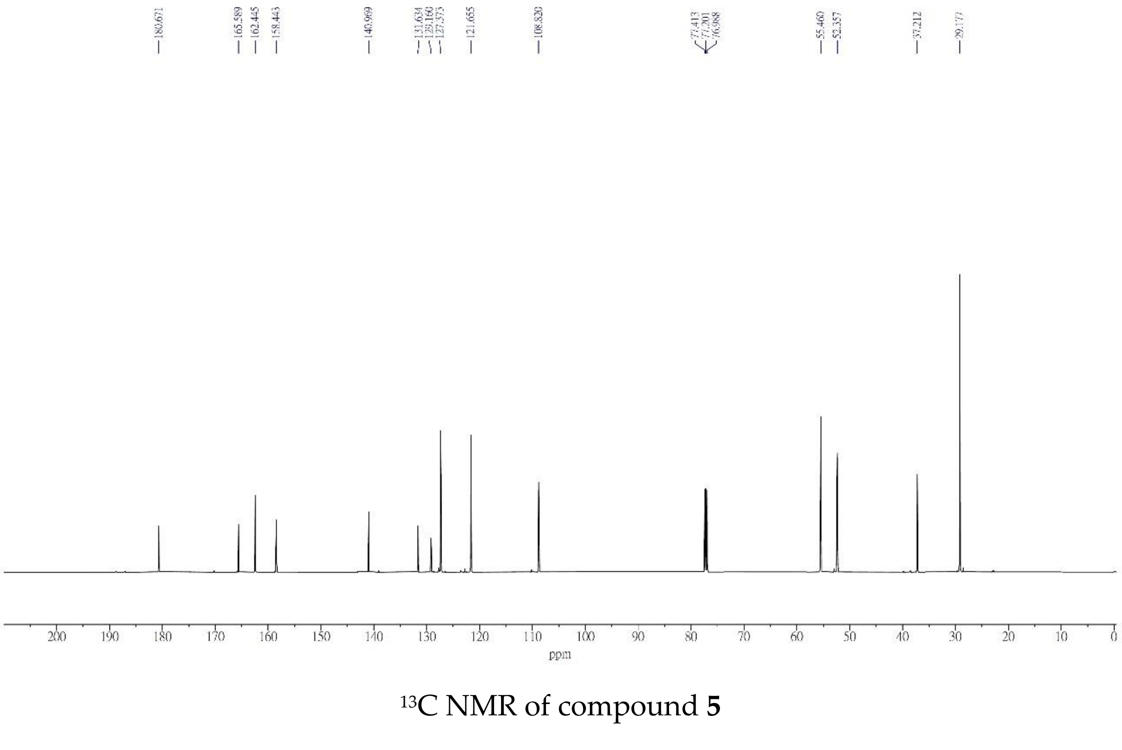

^{13}C NMR of compound **5**

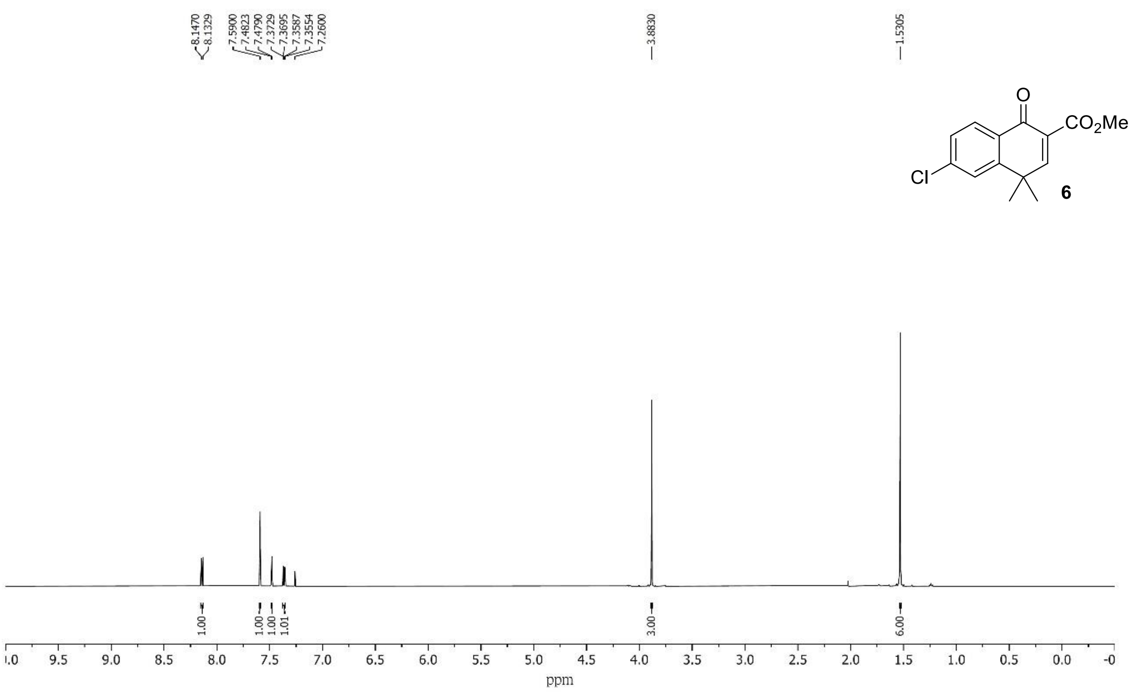

^{1}H NMR of compound **6**

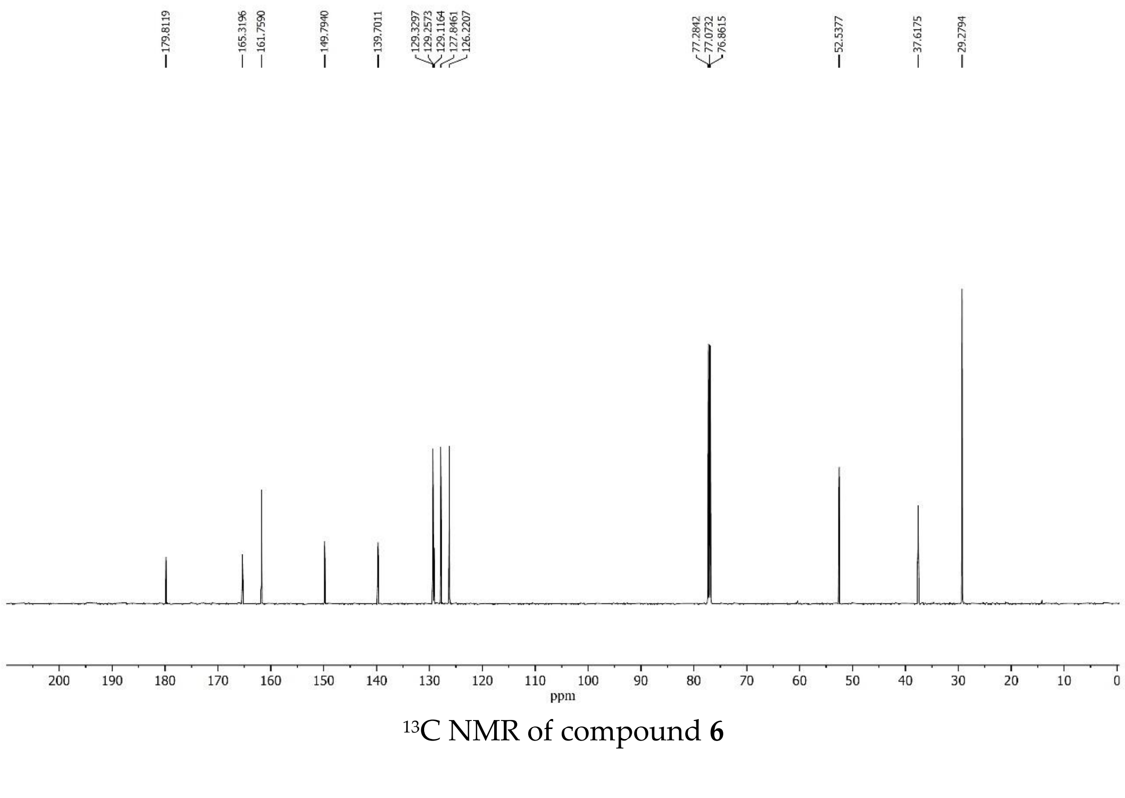

^{13}C NMR of compound **6**

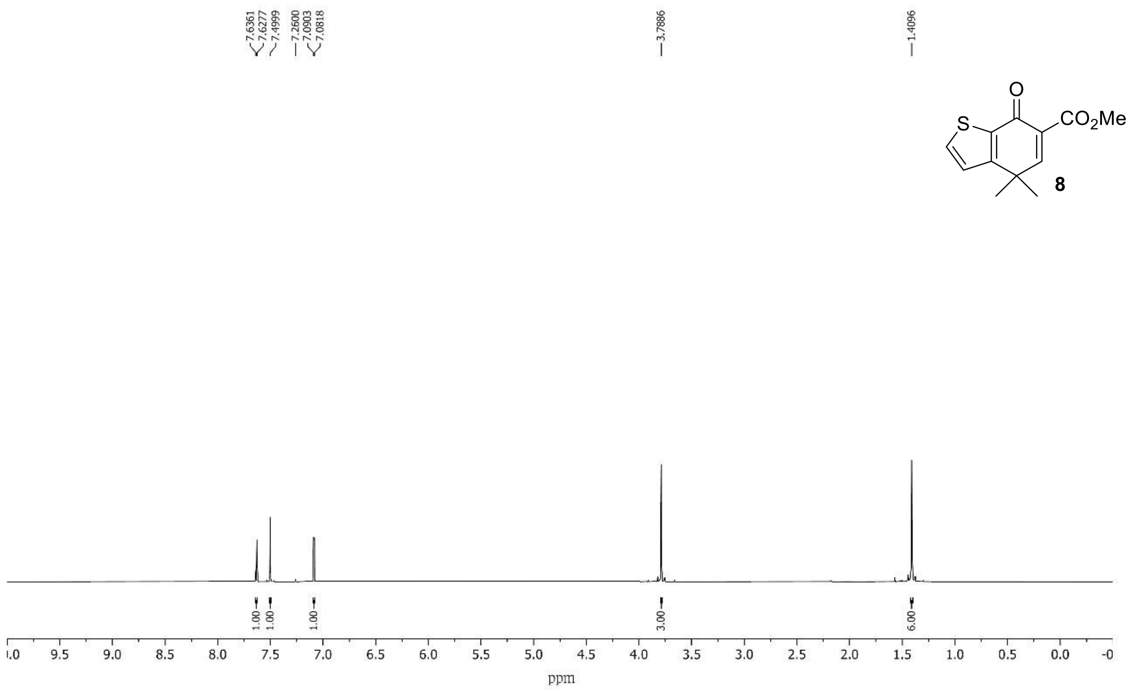

¹H NMR of compound **8**

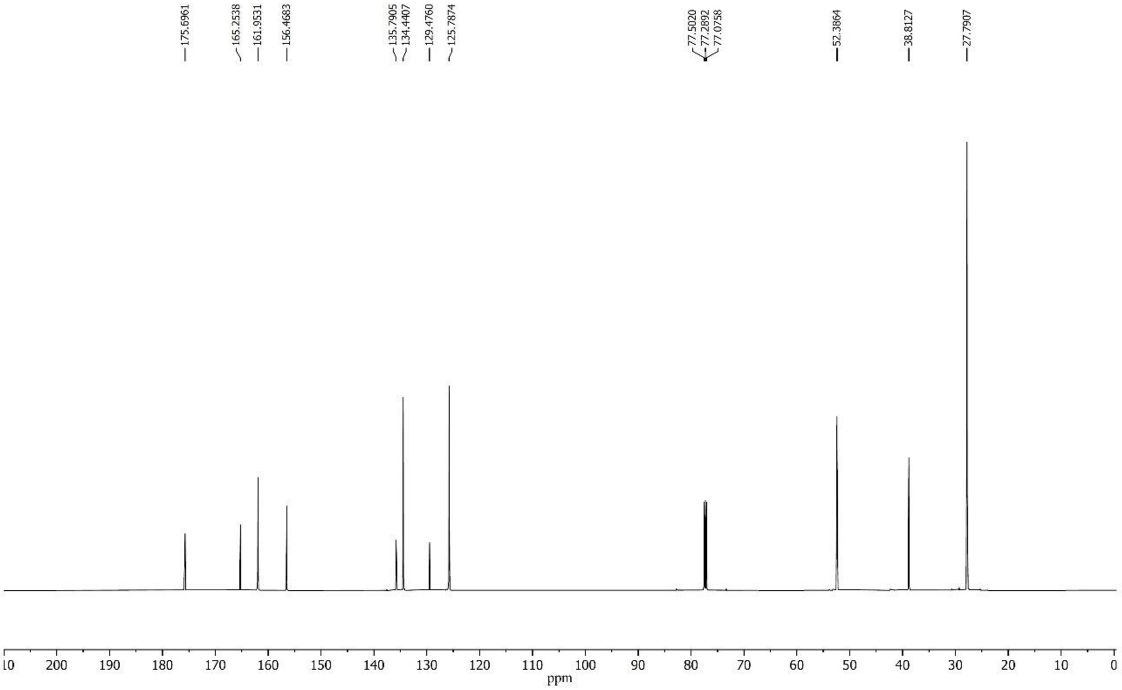

¹³C NMR of compound **8**

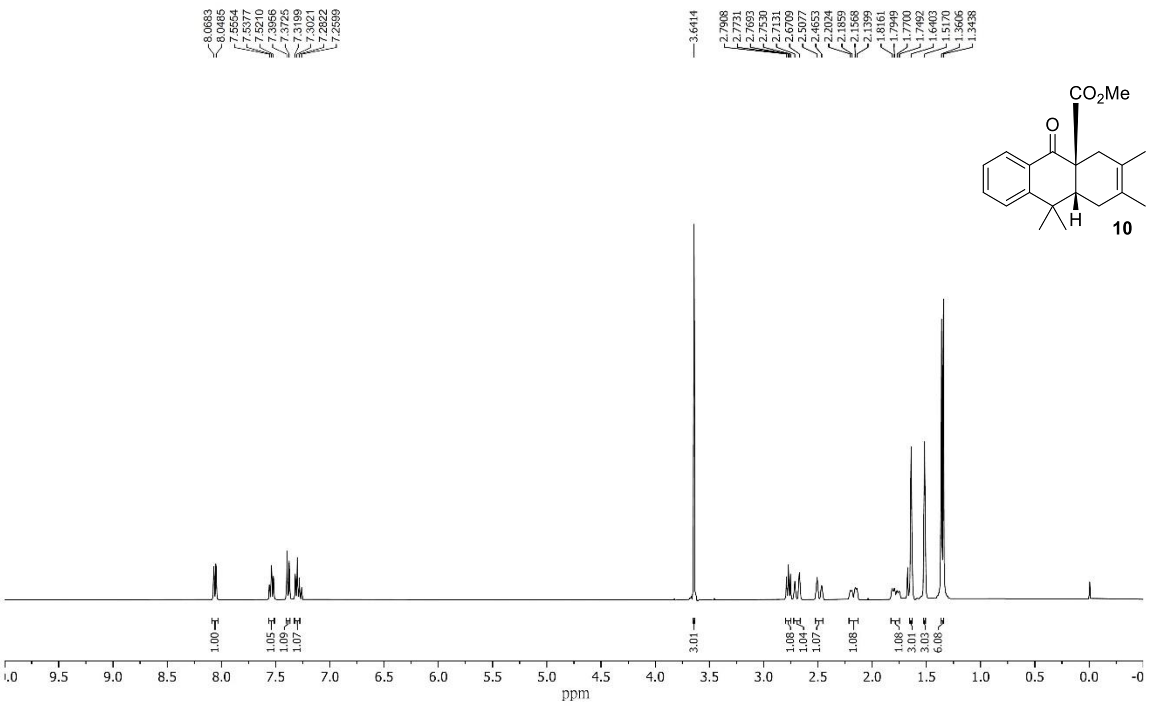

^{1}H NMR spectra of compound **10**

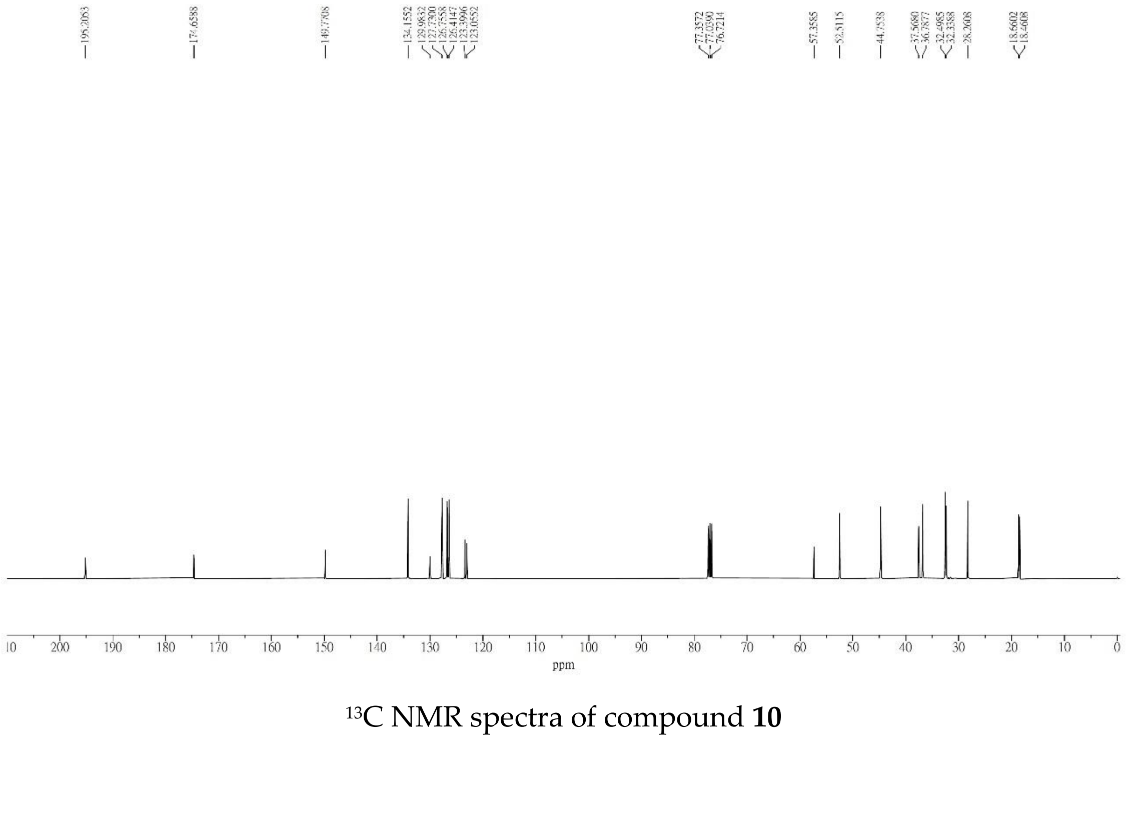

^{13}C NMR spectra of compound **10**

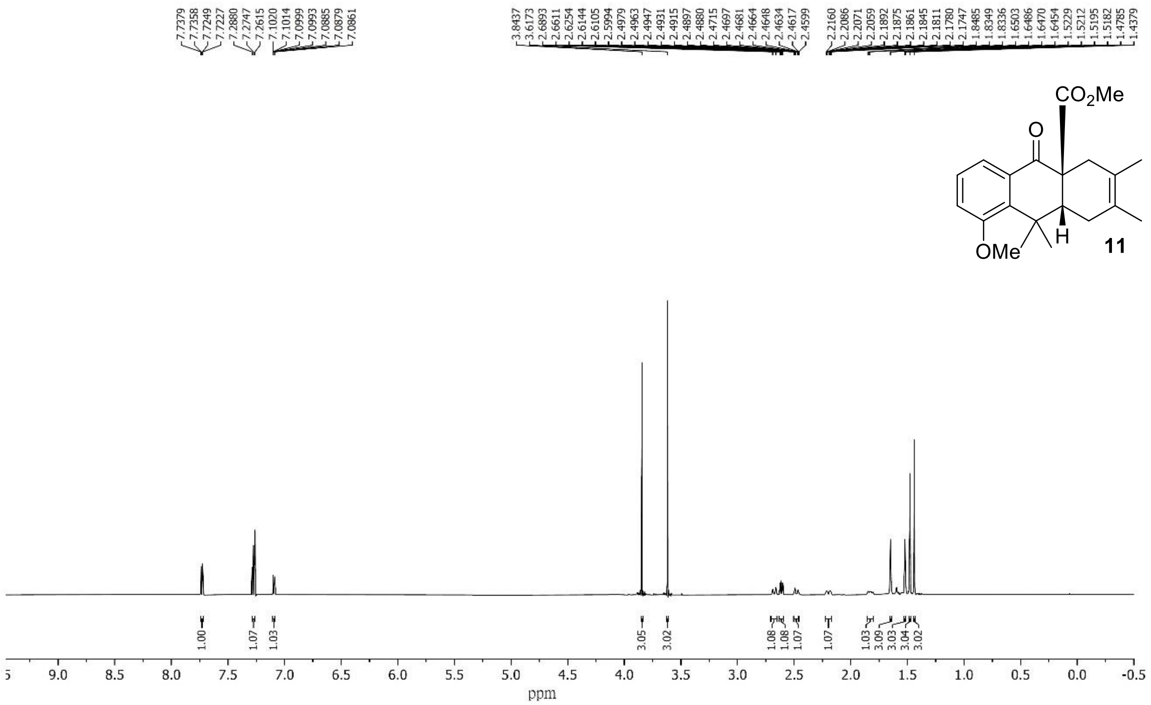

^{1}H NMR of compound **11**

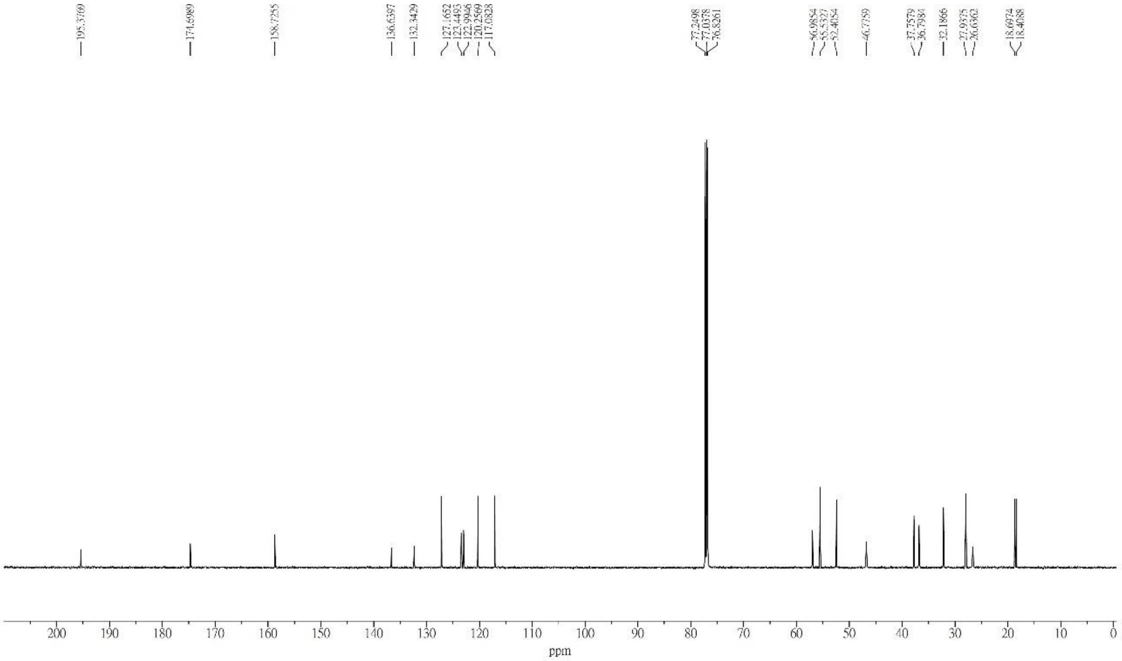

^{13}C NMR of compound **11**

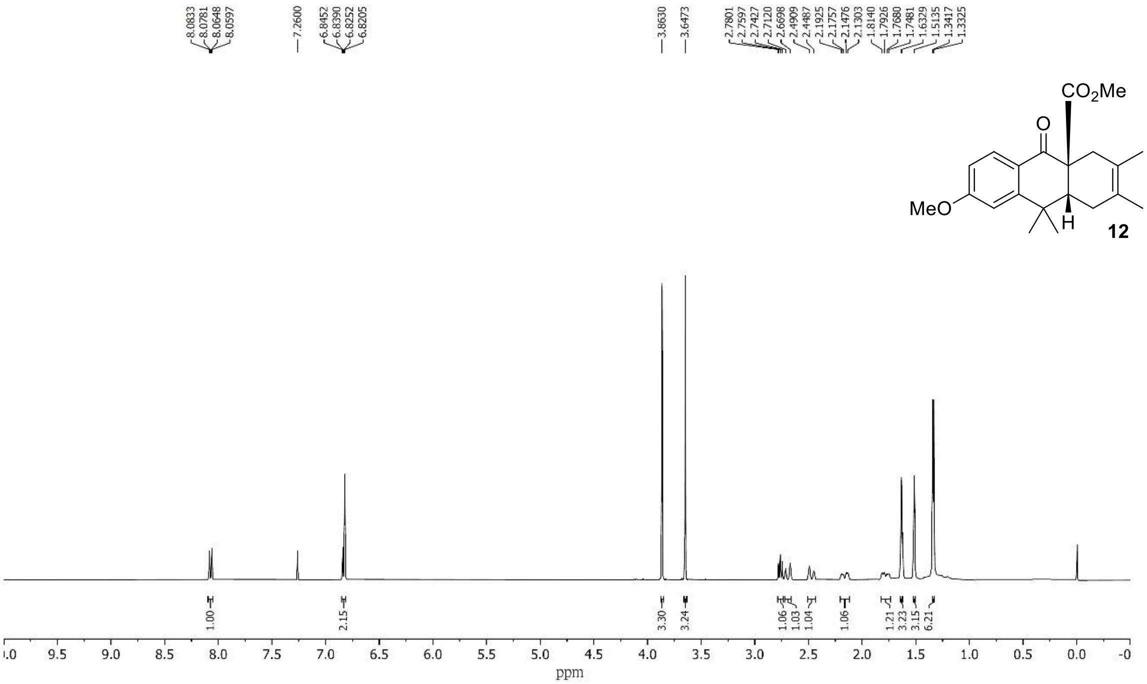

^{1}H NMR of compound **12**

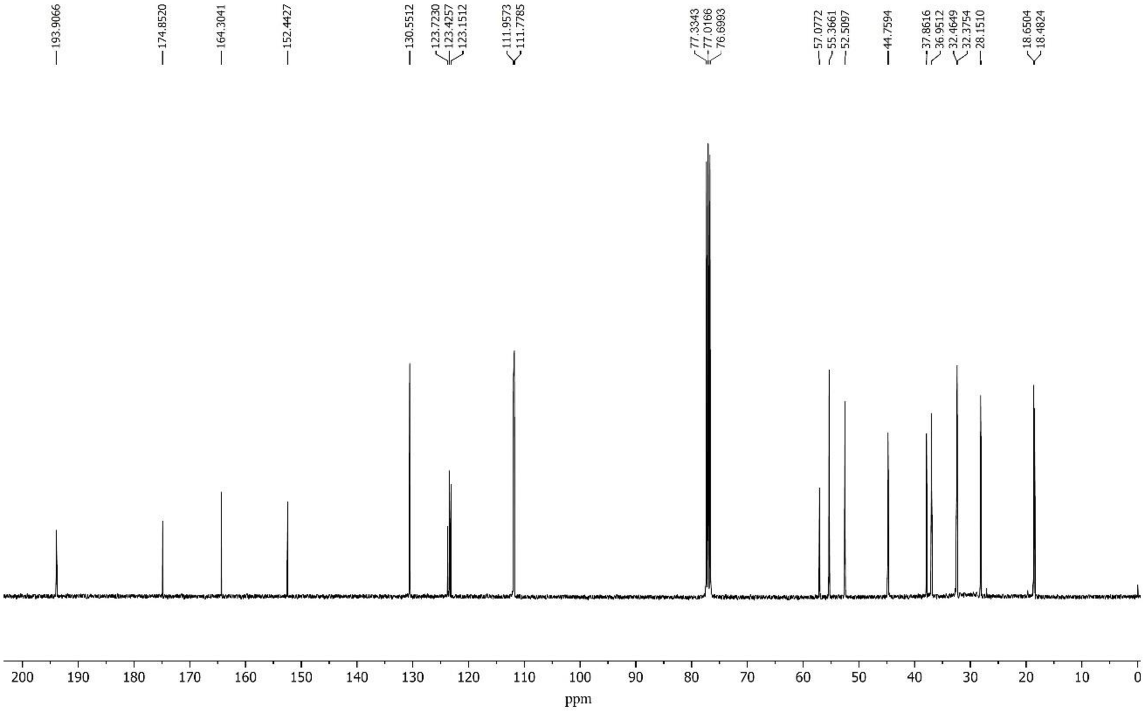

^{13}C NMR of compound **12**

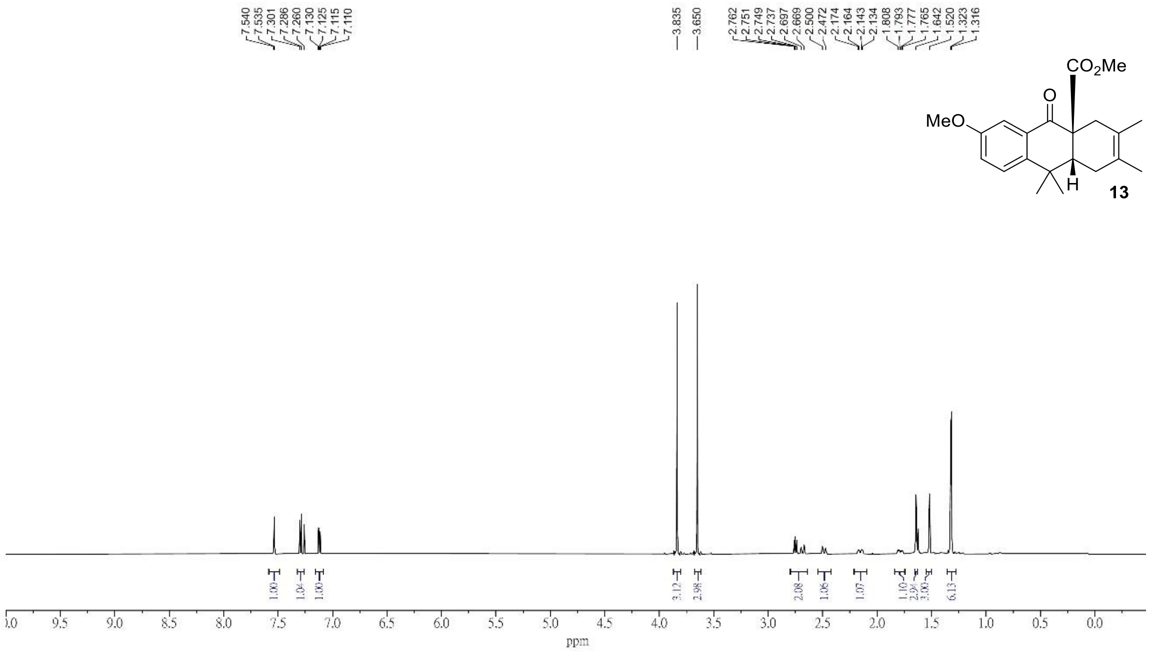

^{1}H NMR of compound **13**

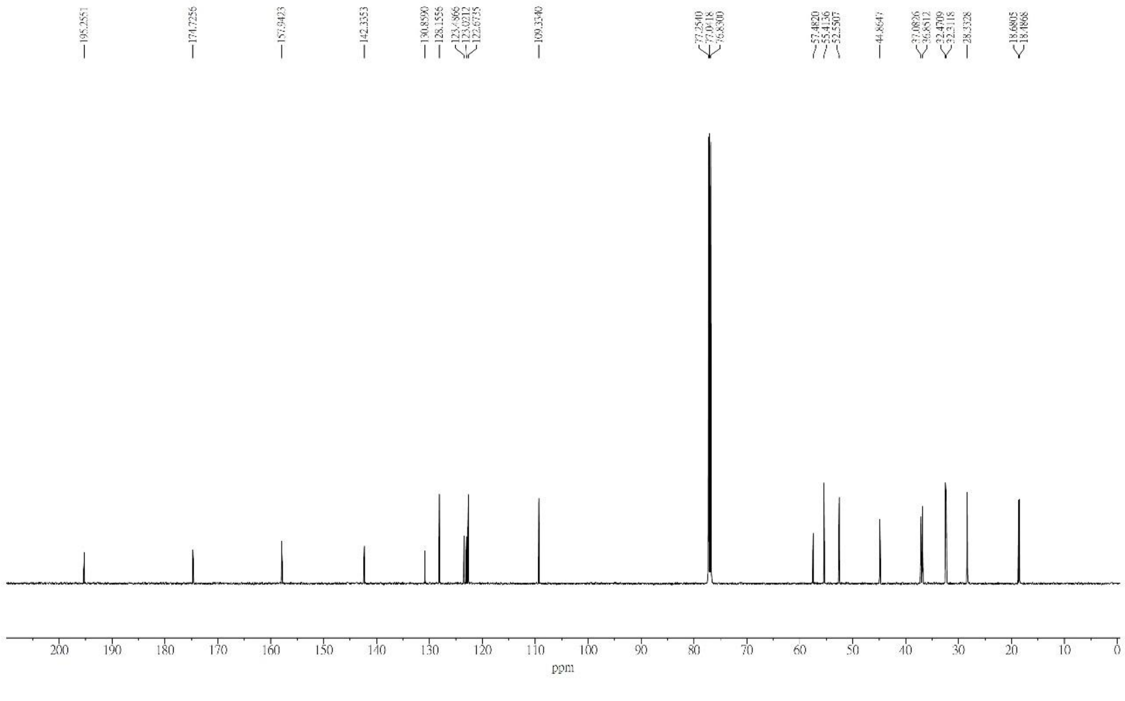

^{13}C NMR of compound **13**

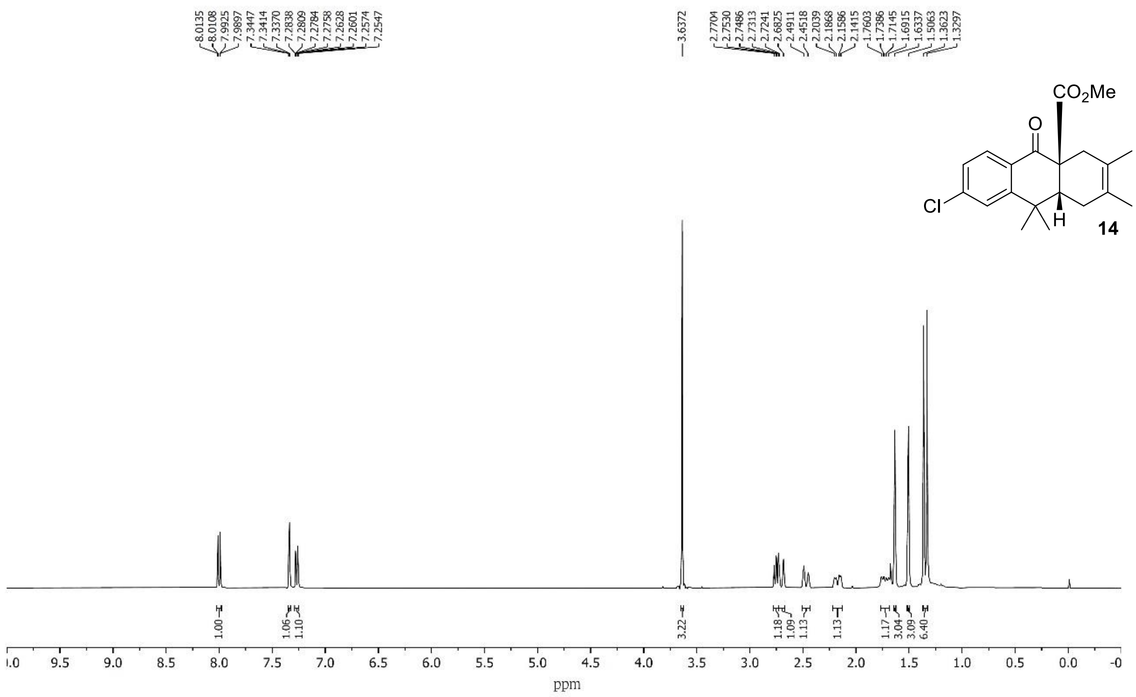

¹H NMR of compound **14**

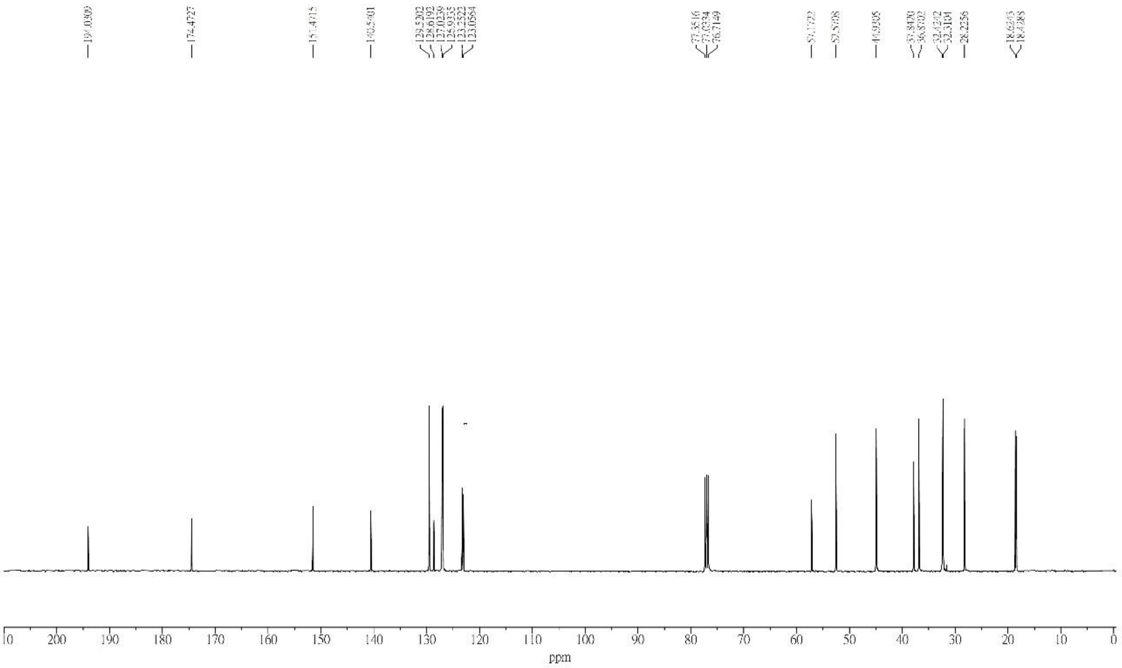

¹³C NMR of compound **14**

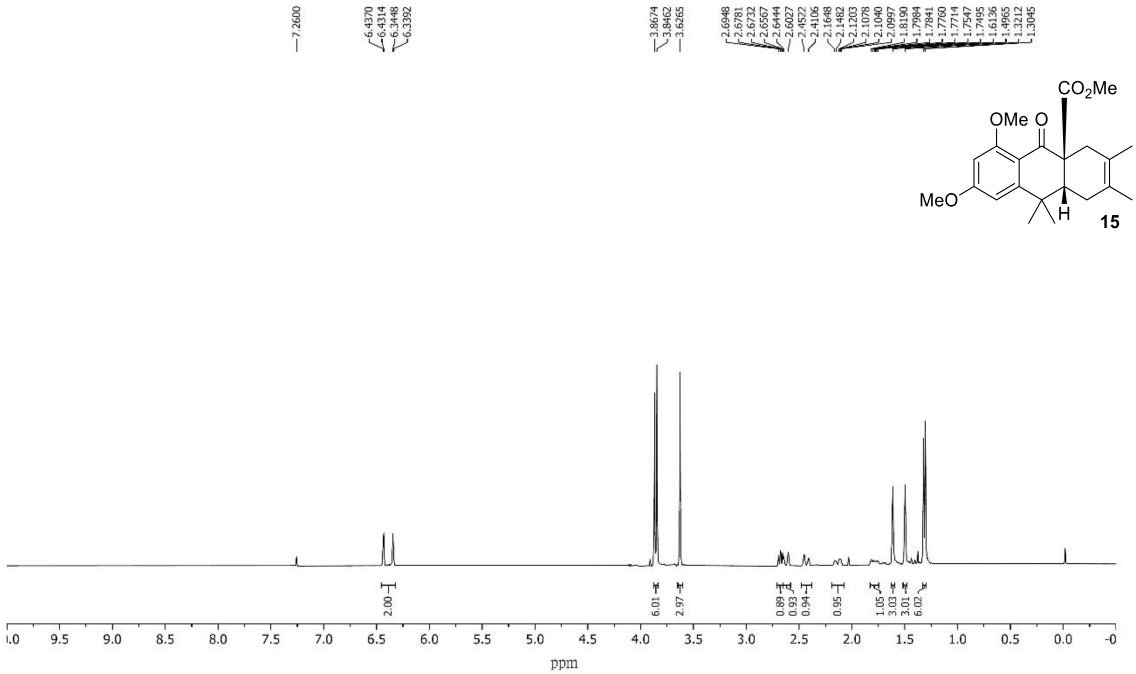

^{1}H NMR of compound **15**

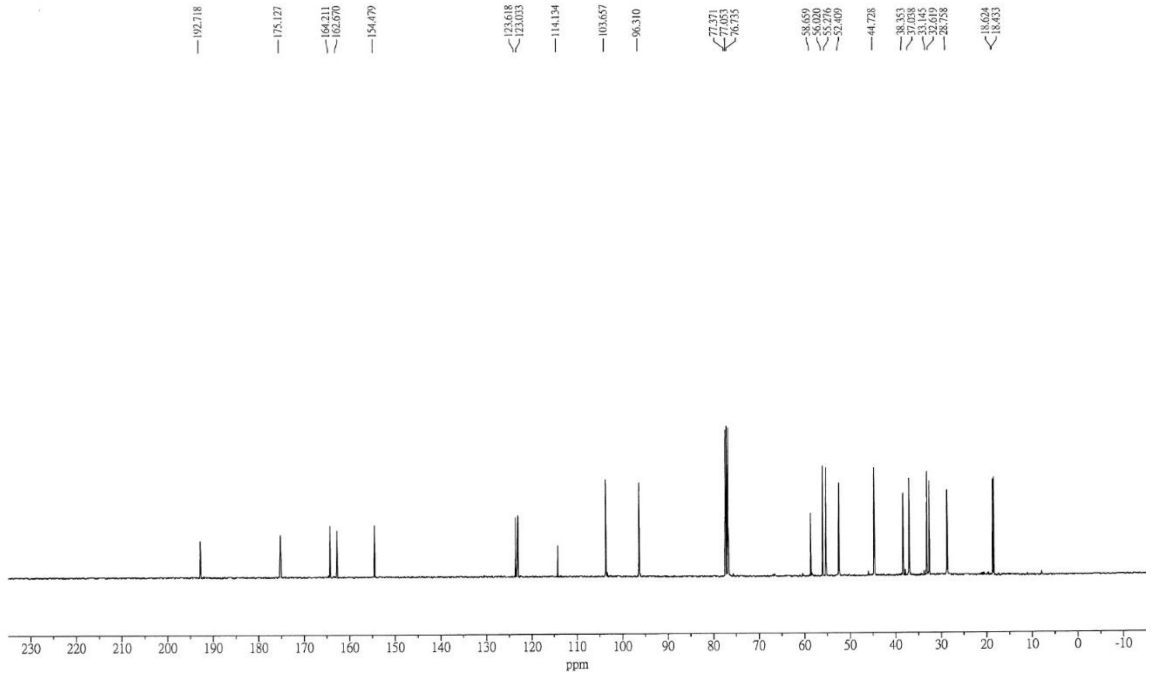

^{13}C NMR of compound **15**

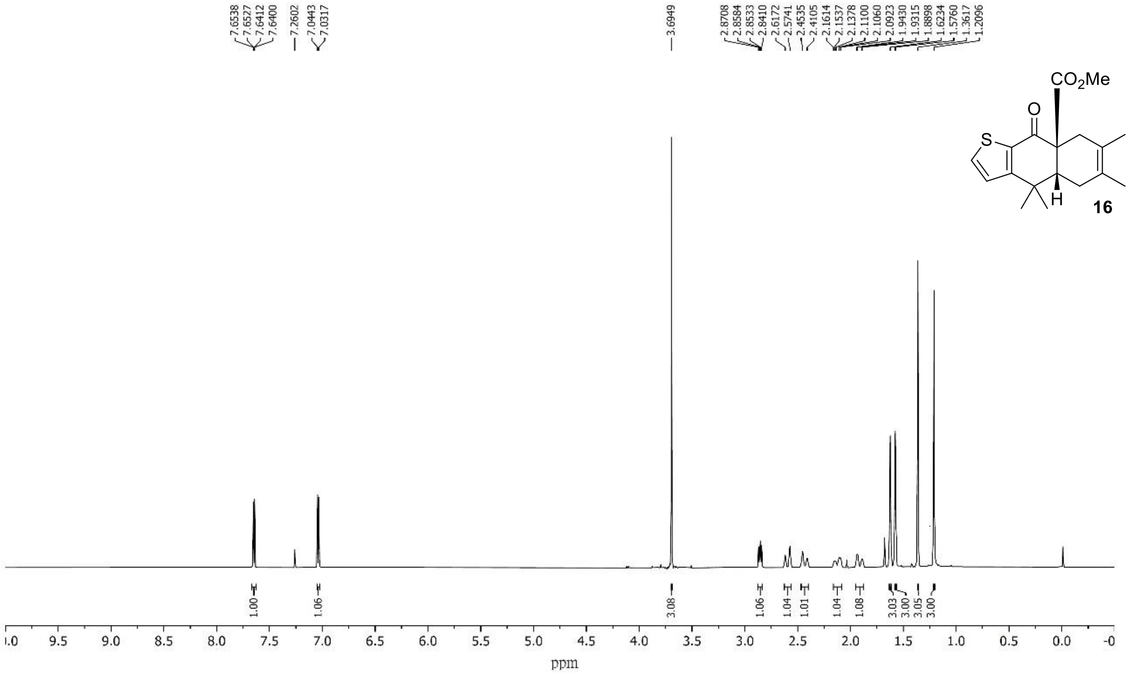

^{1}H NMR of compound **16**

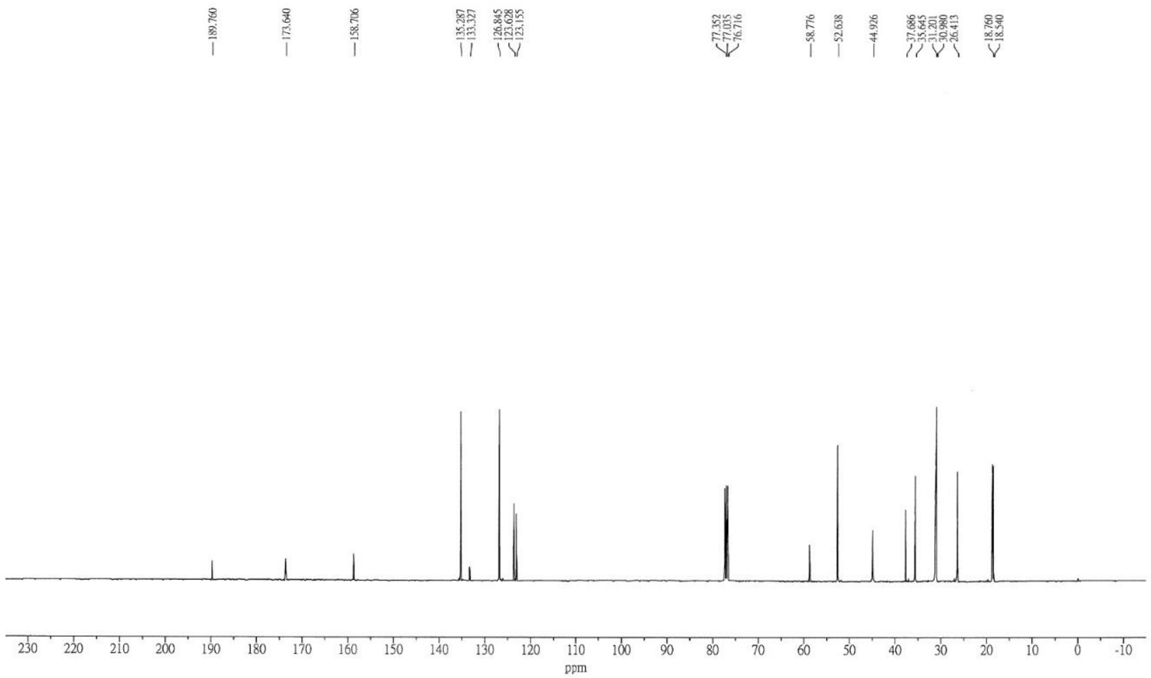

^{13}C NMR of compound **16**

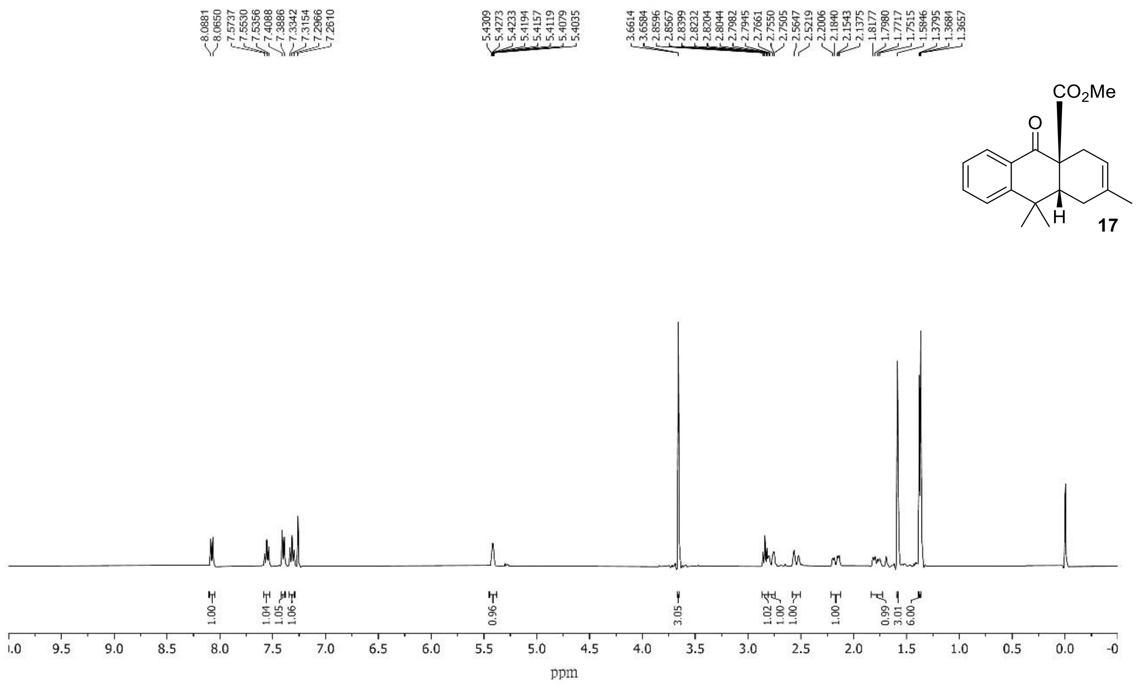

^{1}H NMR of compound **17**

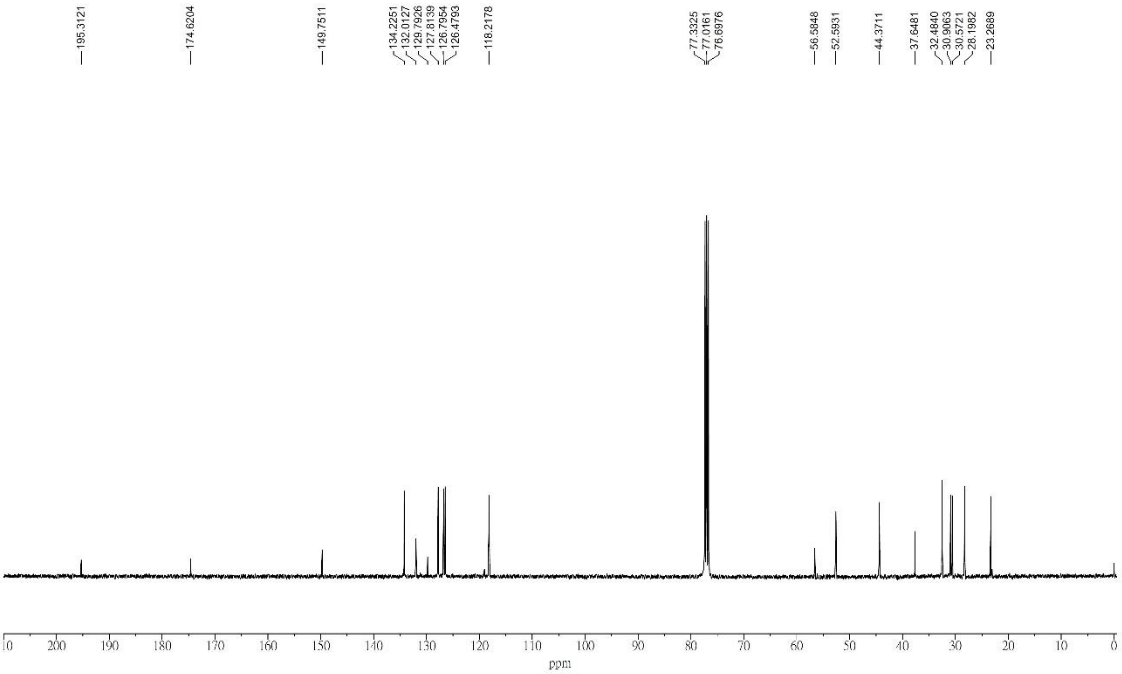

^{13}C NMR of compound **17**

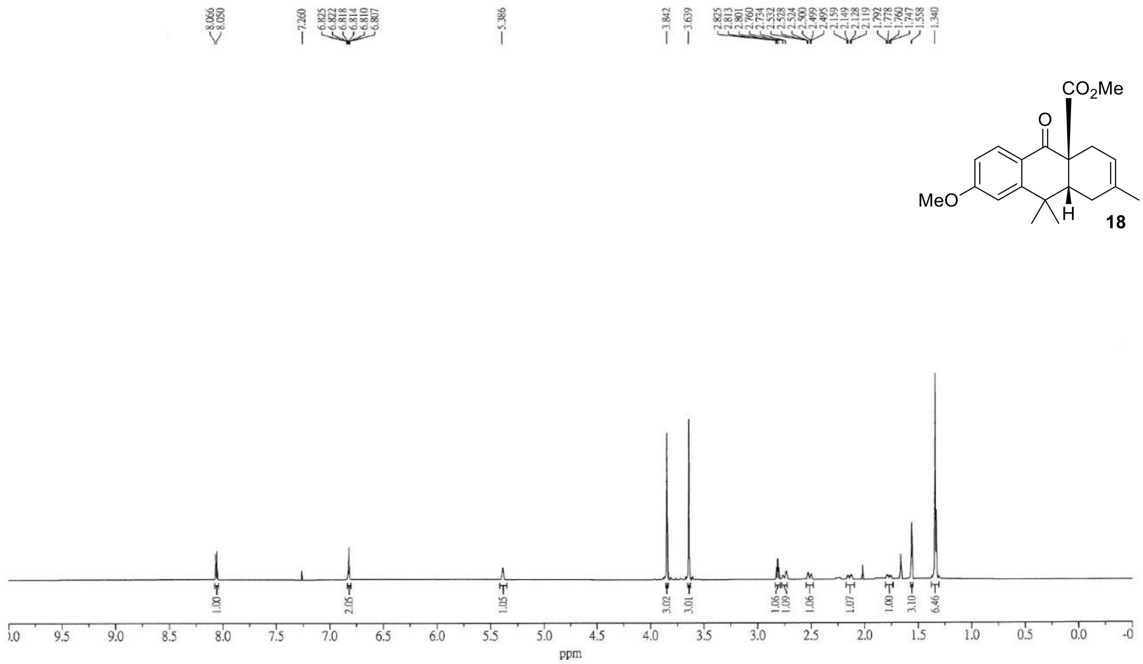

^{1}H NMR of compound **18**

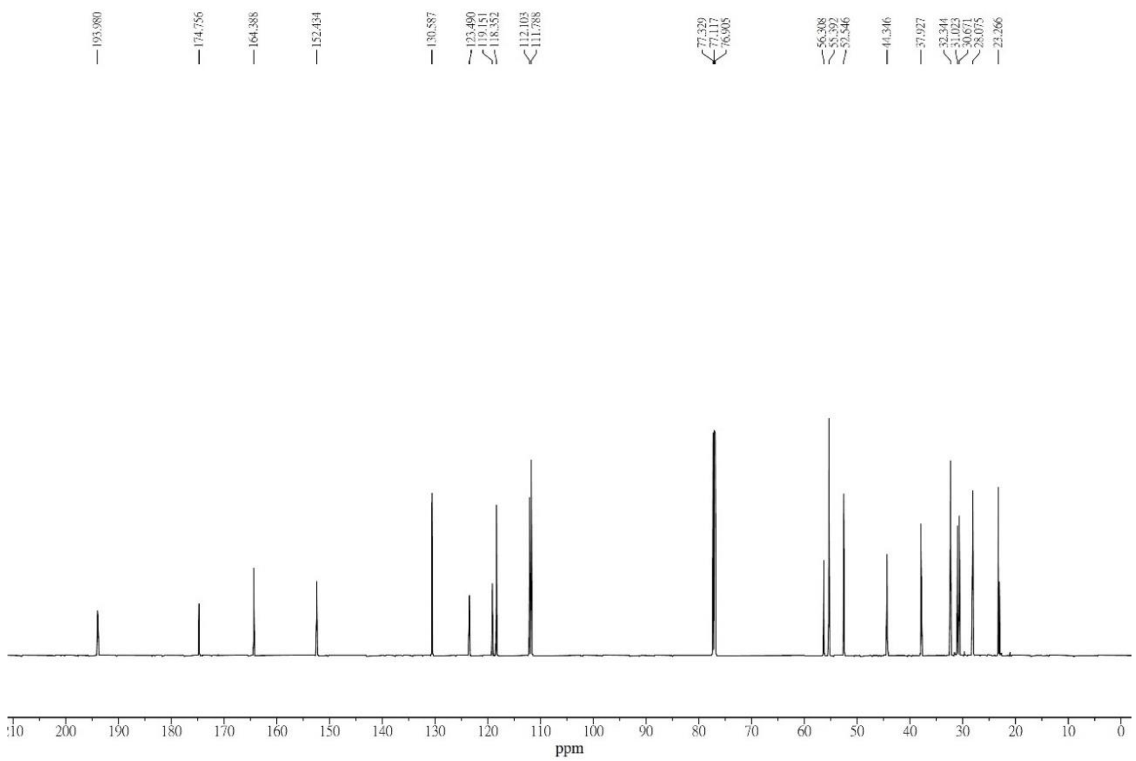

^{13}C NMR of compound **18**

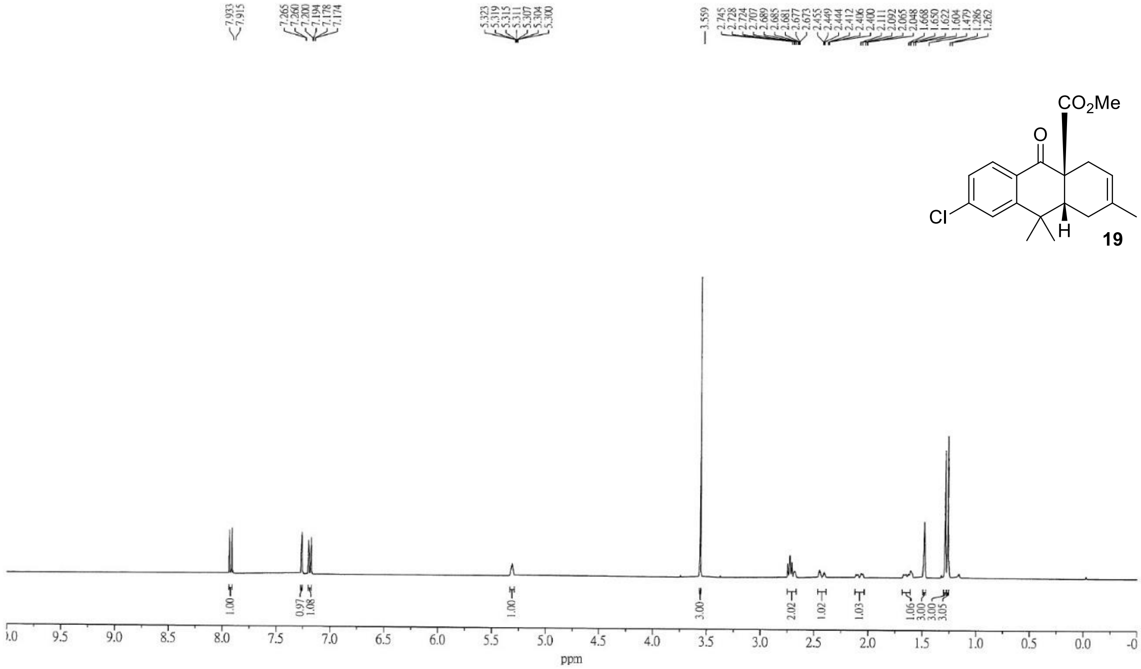

^{1}H NMR of compound **19**

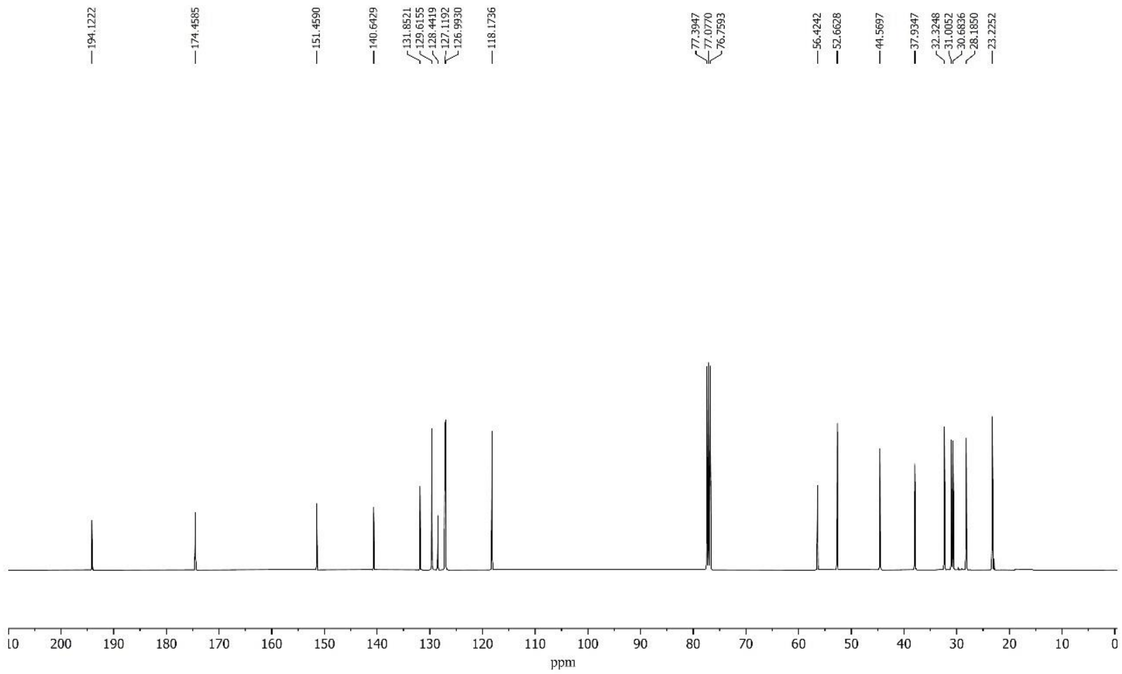

^{13}C NMR of compound **19**

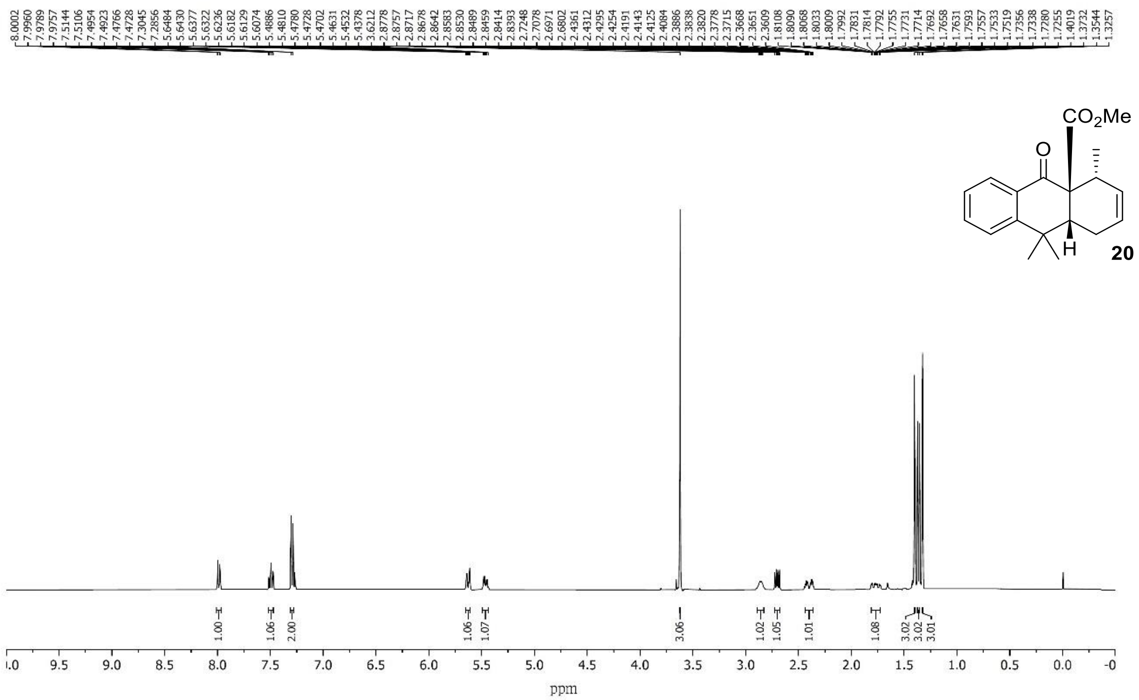

^{1}H NMR of compound **20**

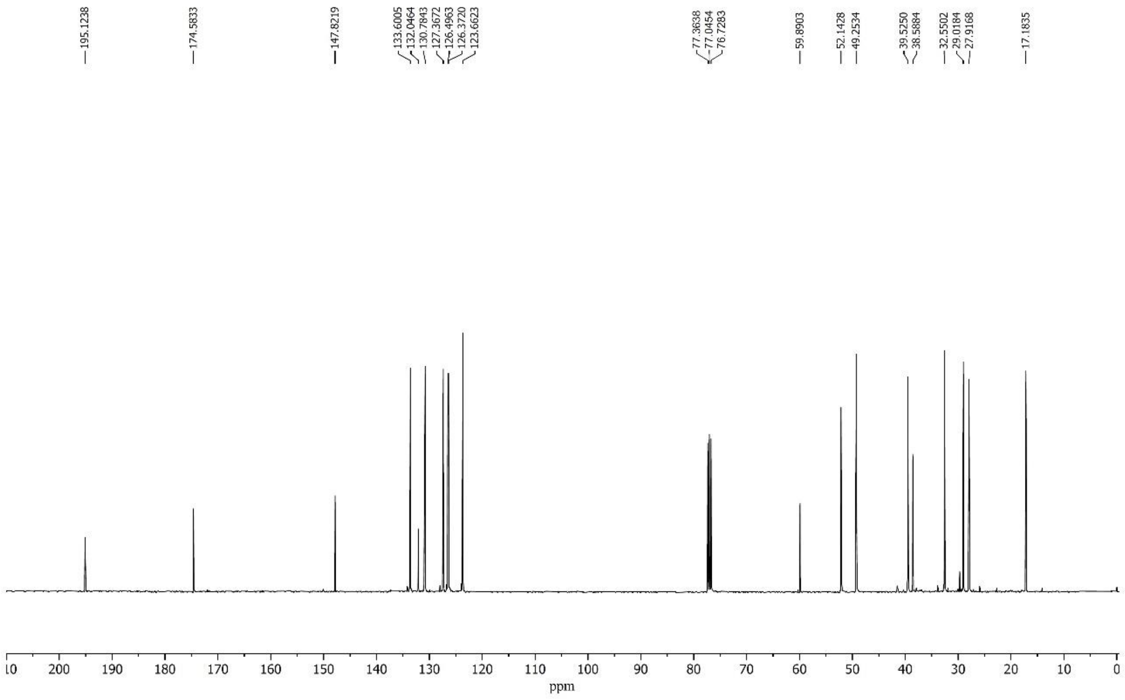

^{13}C NMR of compound **20**

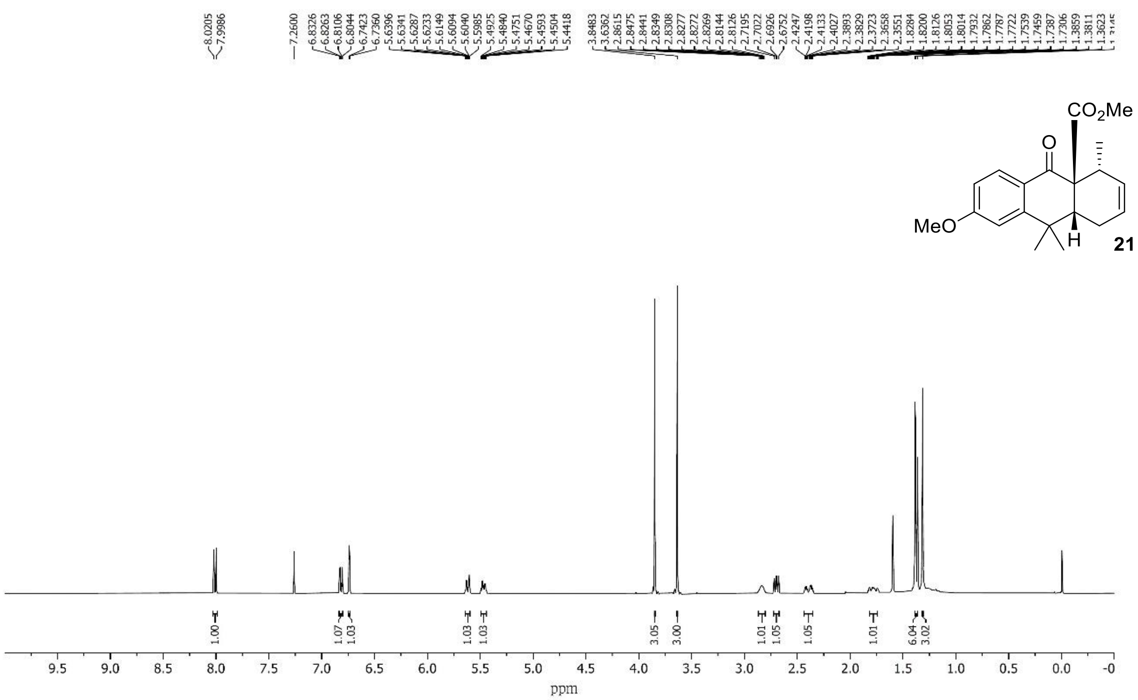

^{1}H NMR of compound **21**

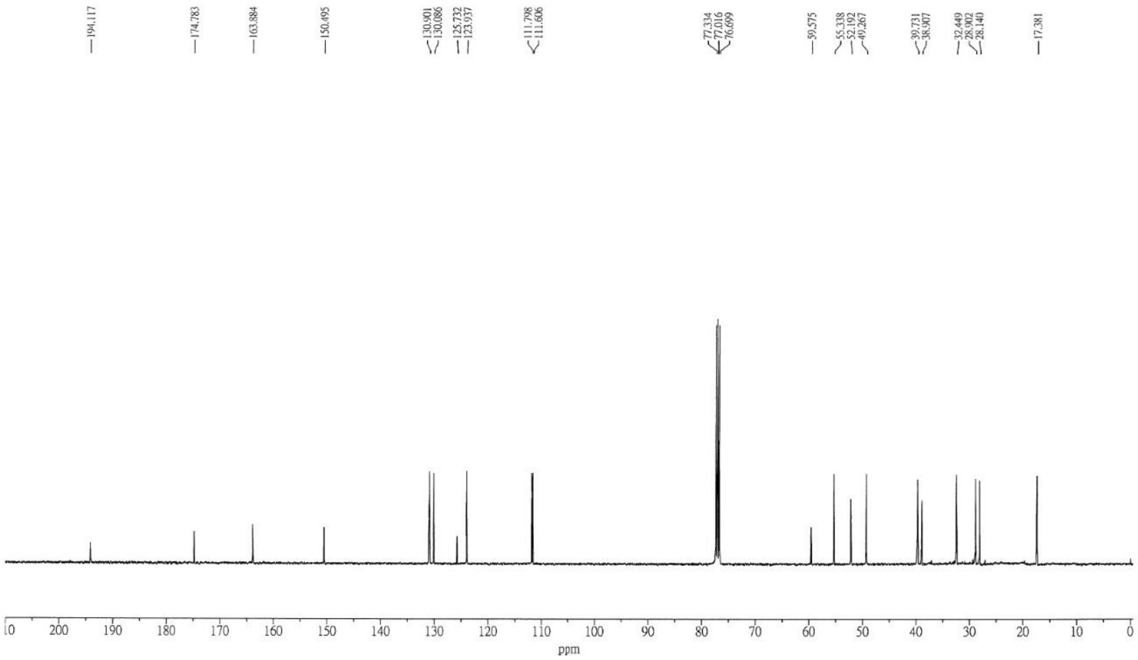

^{13}C NMR of compound **21**

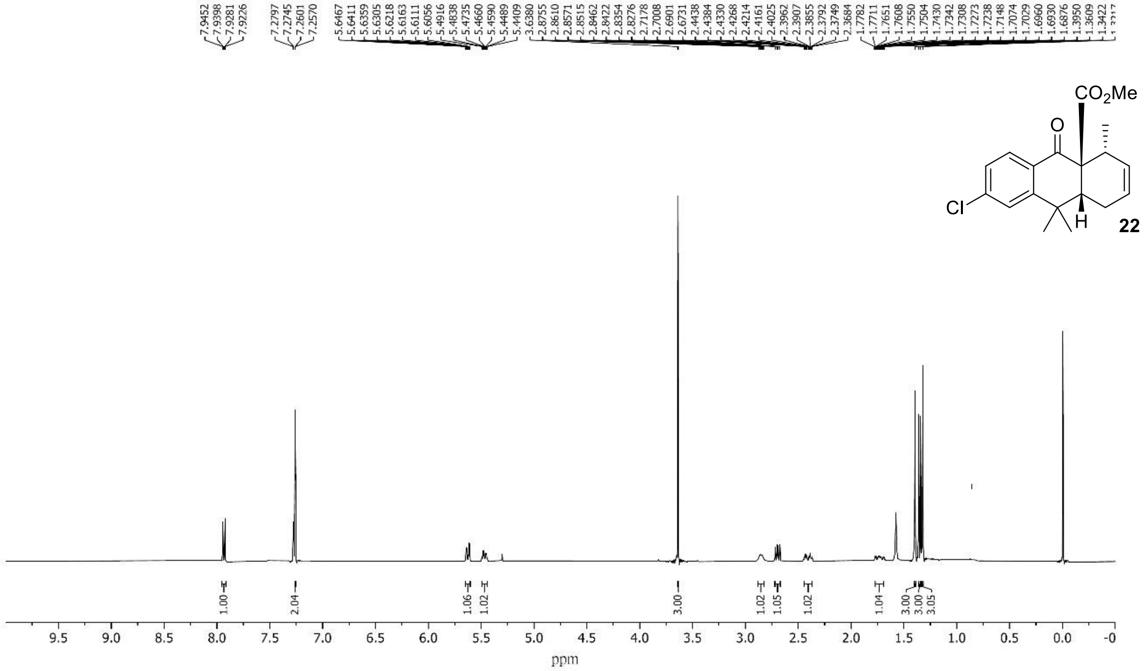

^{1}H NMR of compound **22**

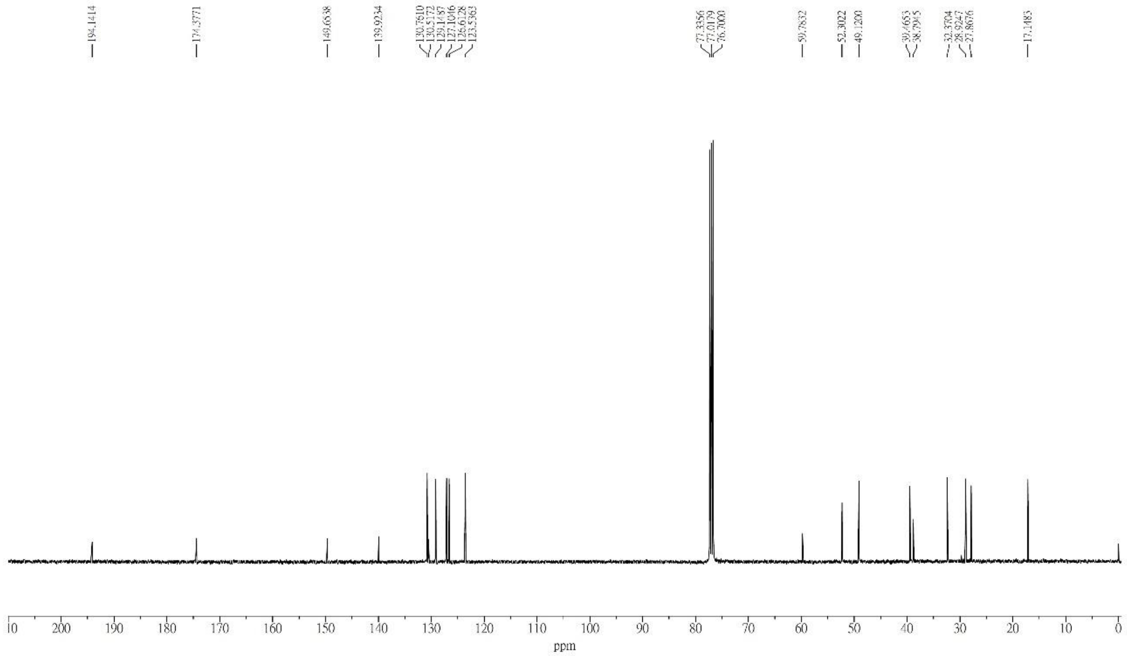

^{13}C NMR of compound **22**

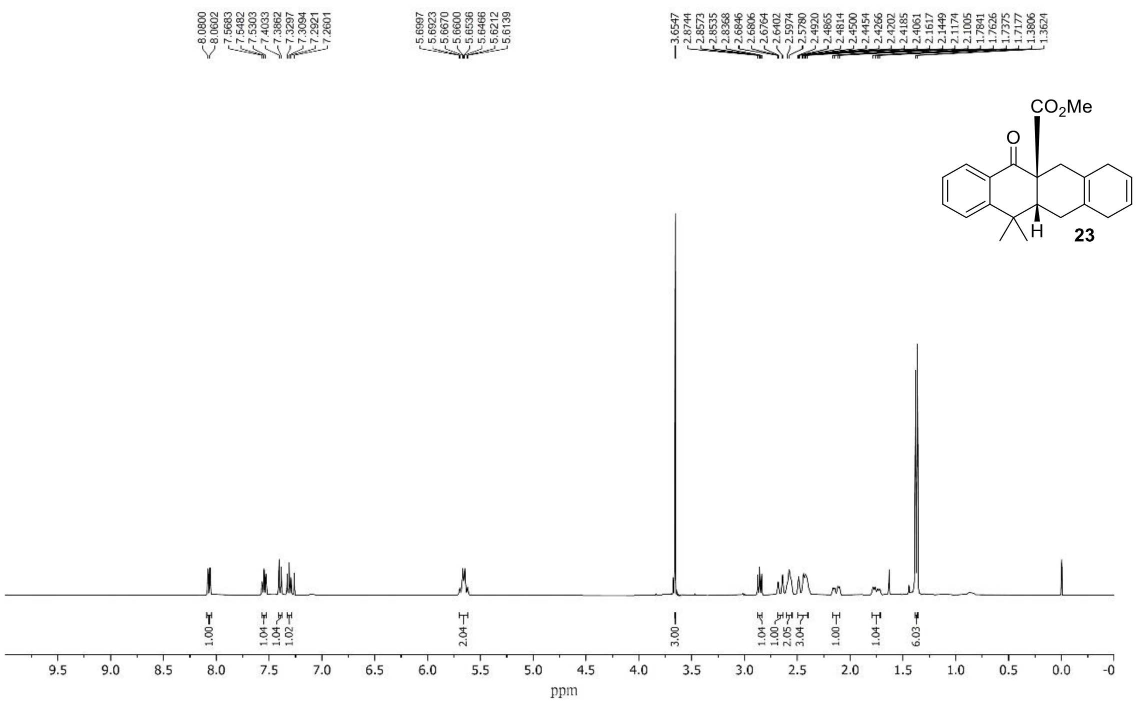

^{1}H NMR of compound **23**

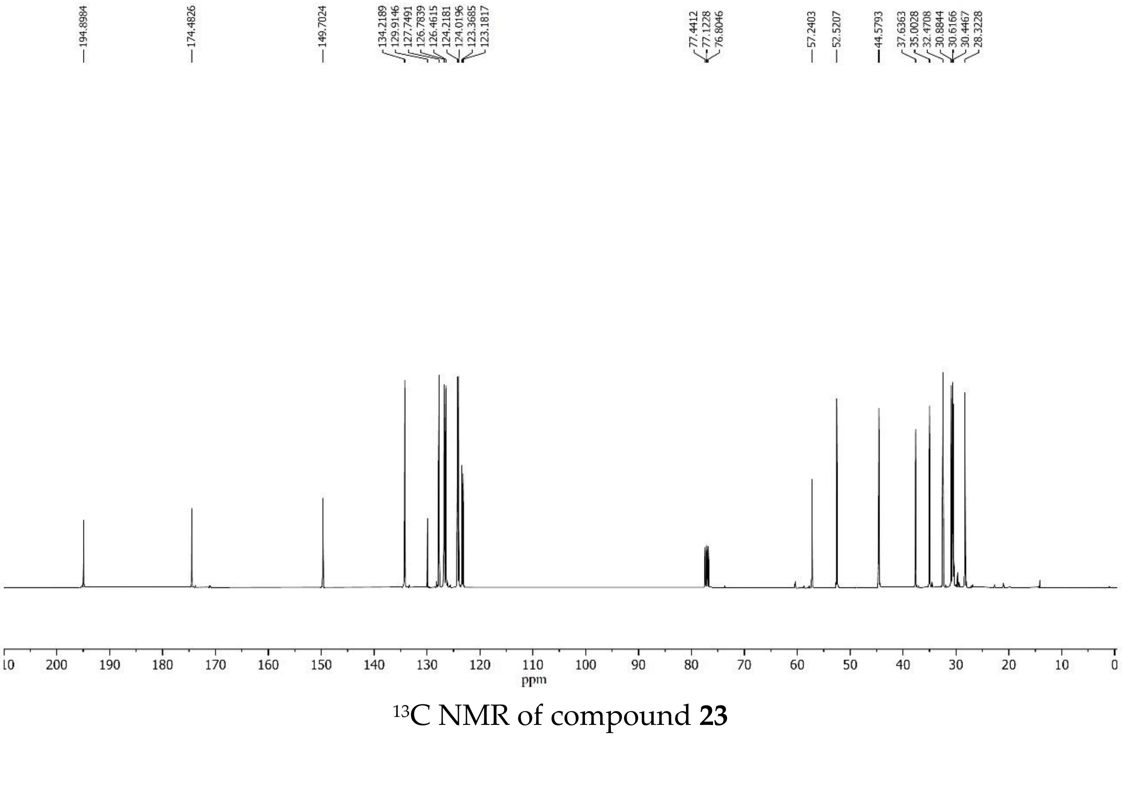

^{13}C NMR of compound **23**

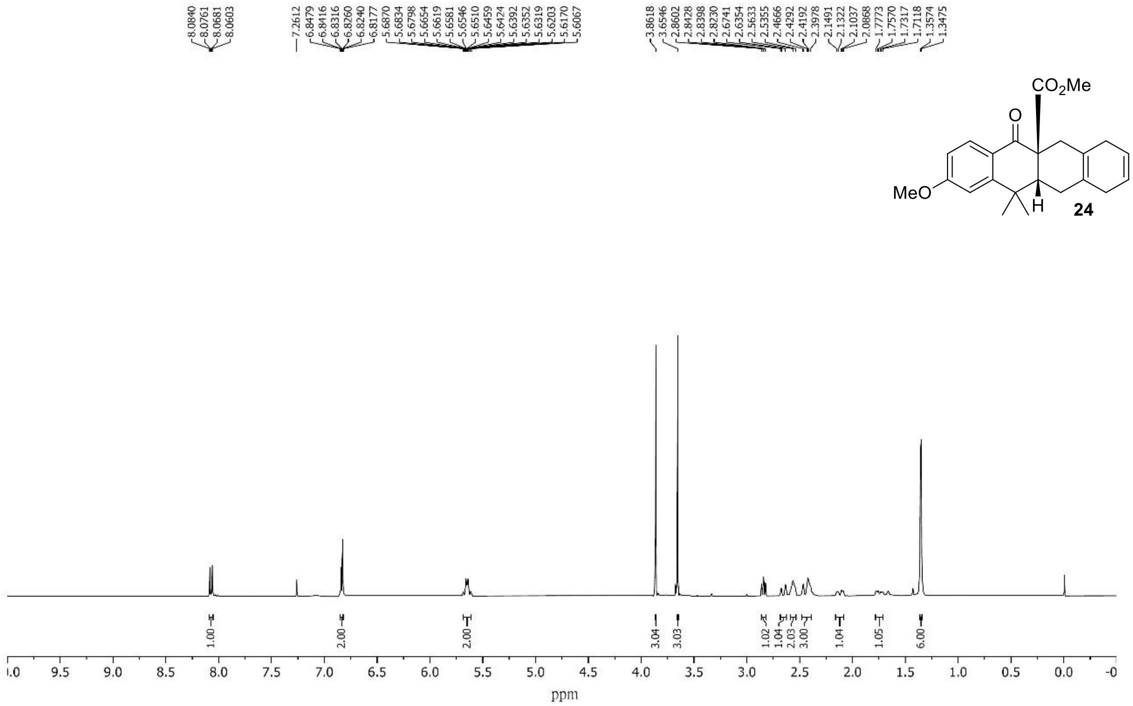

^{1}H NMR of compound **24**

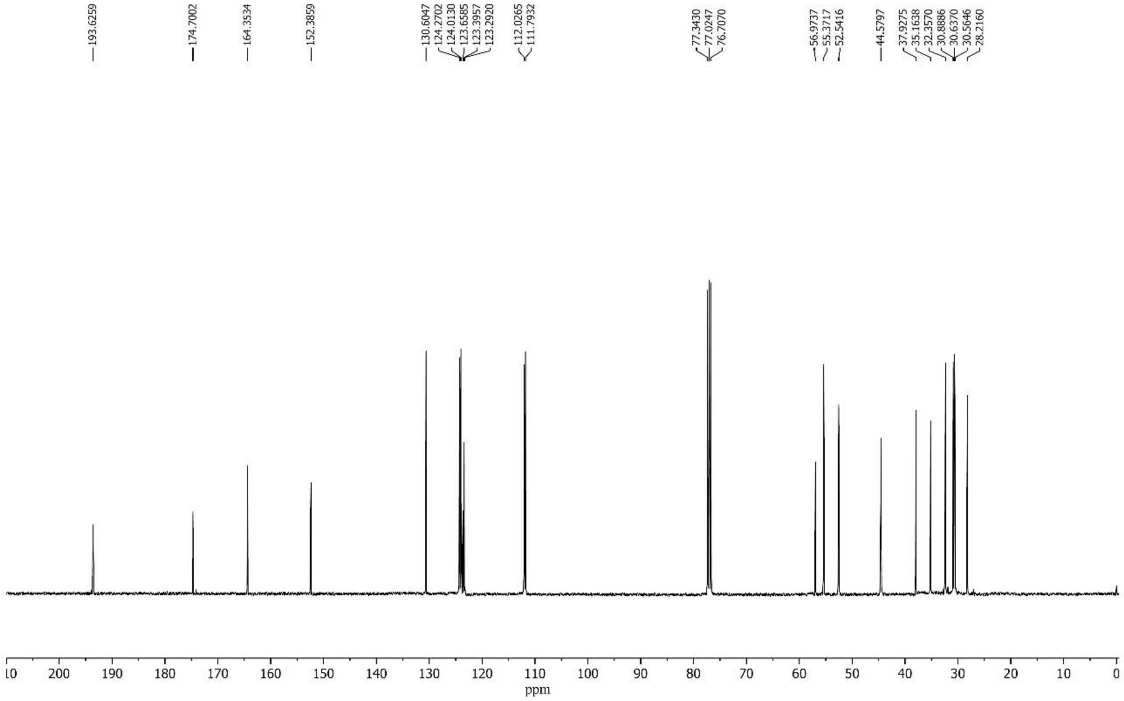

^{13}C NMR of compound **24**

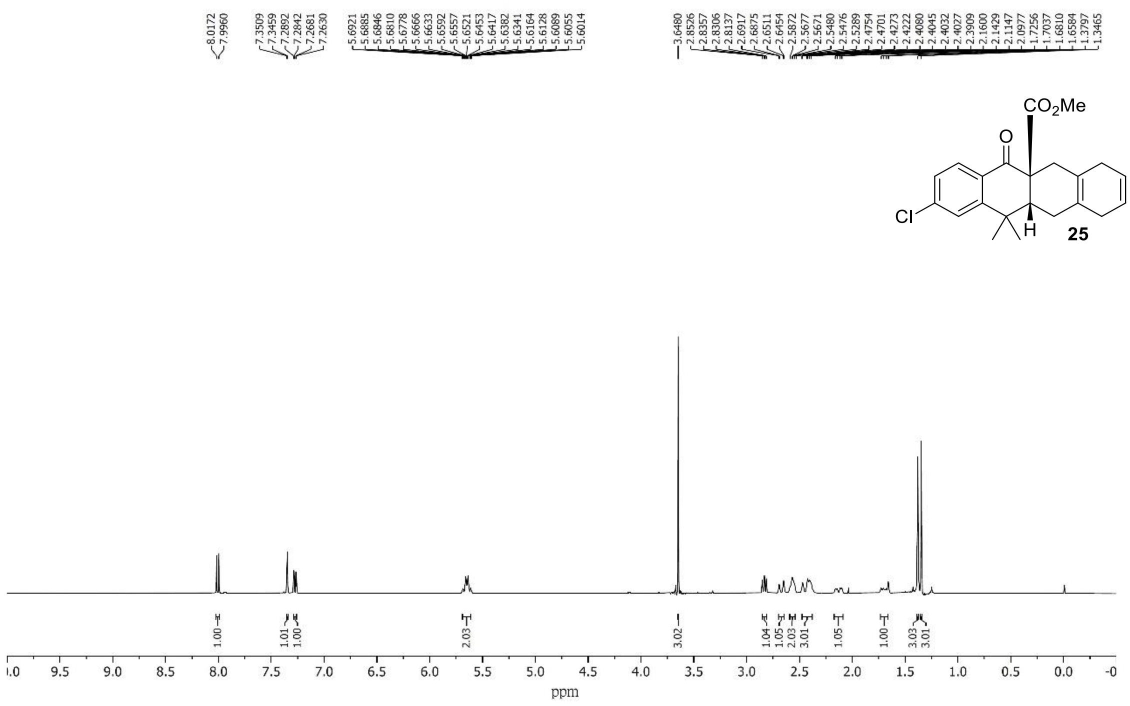

^{1}H NMR of compound **25**

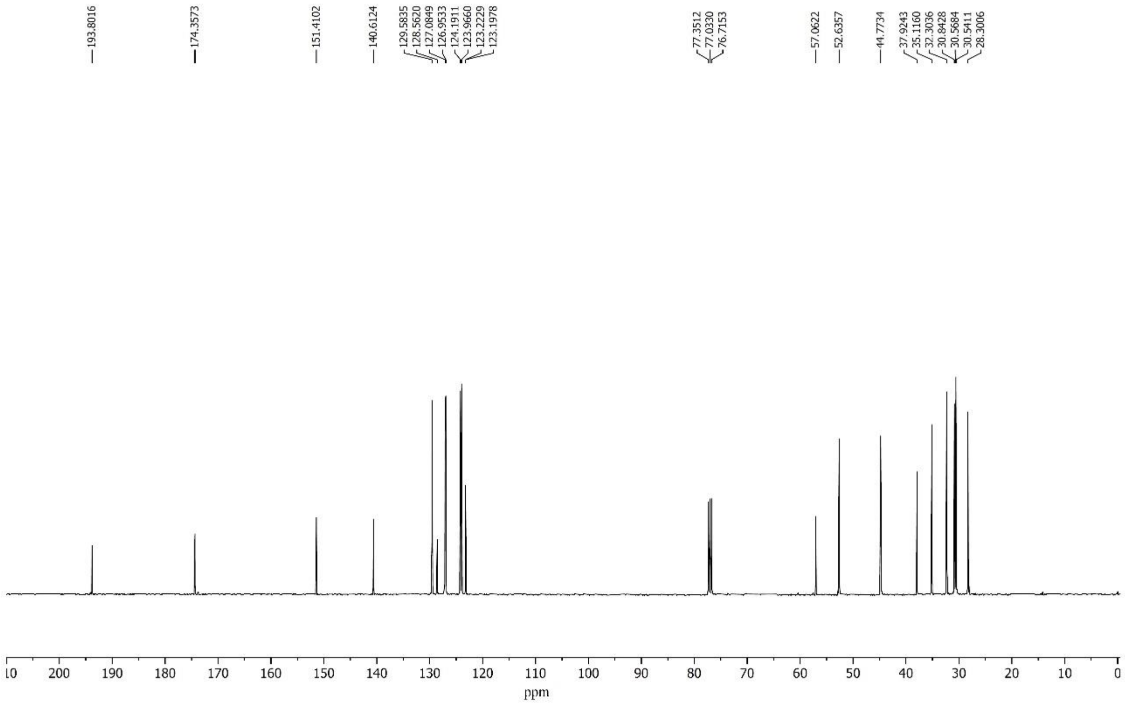

^{13}C NMR of compound **25**

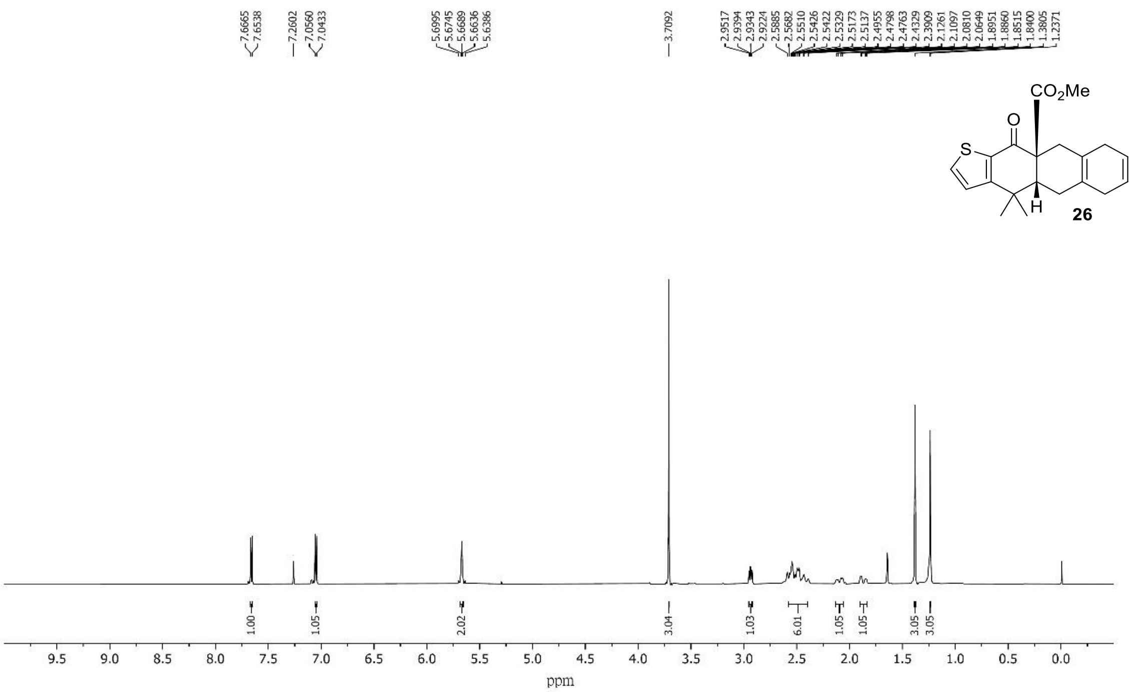

^{1}H NMR of compound **26**

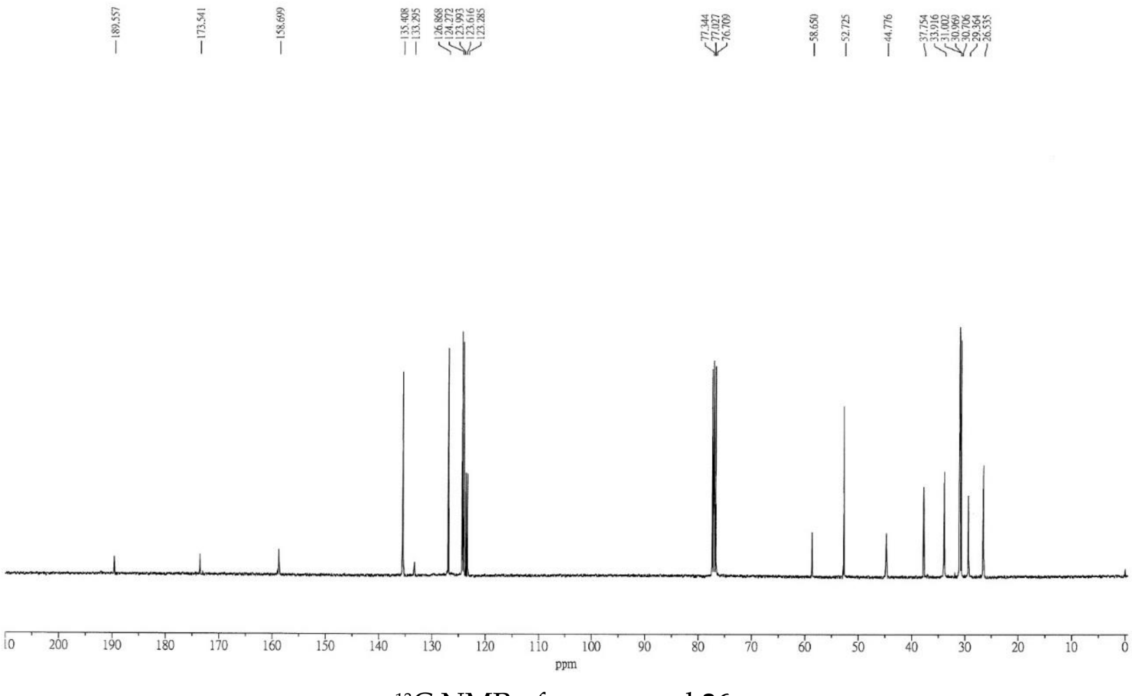

^{13}C NMR of compound **26**

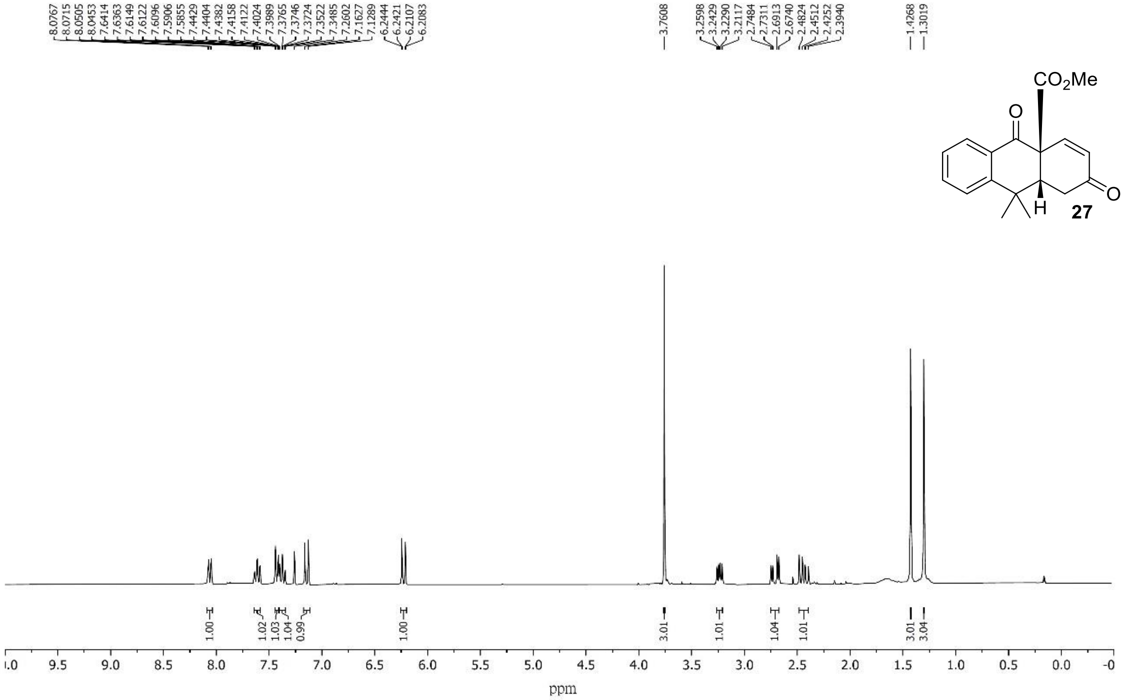

^{1}H NMR of compound **27**

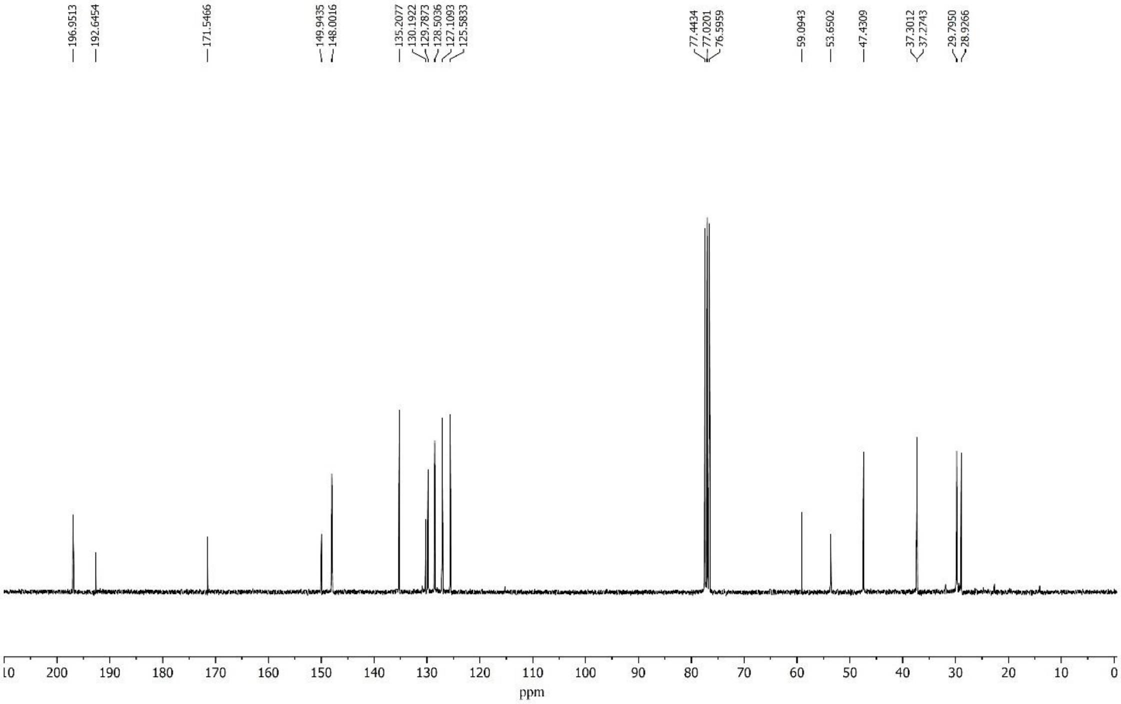

^{13}C NMR of compound **27**

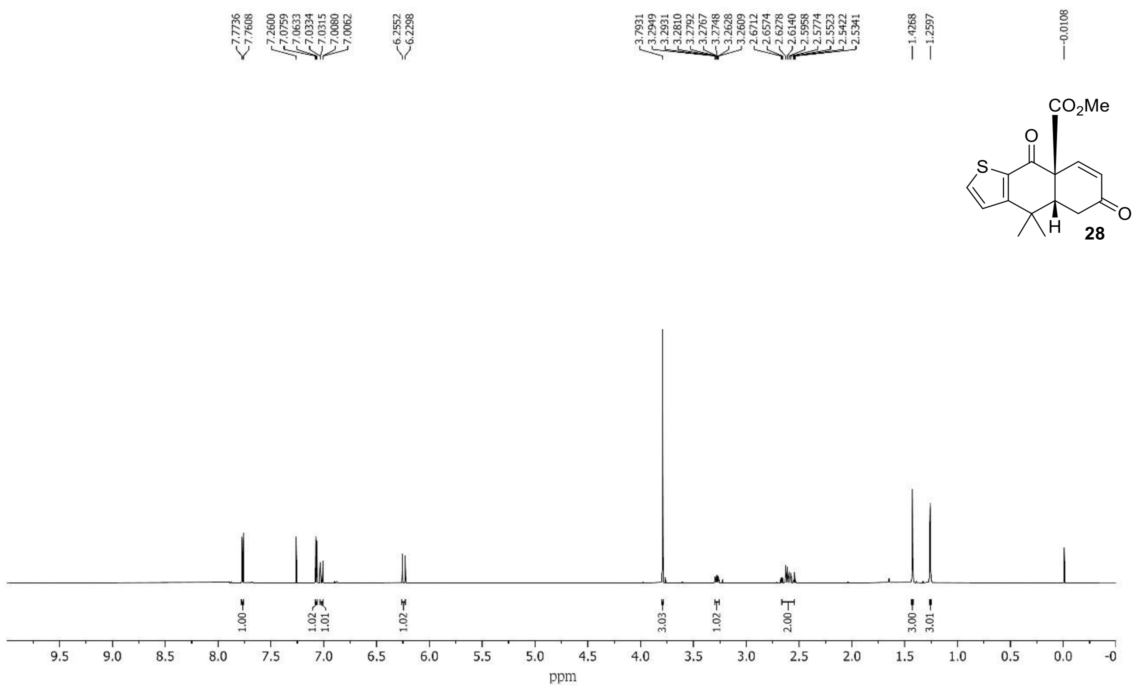

^{1}H NMR of compound **28**

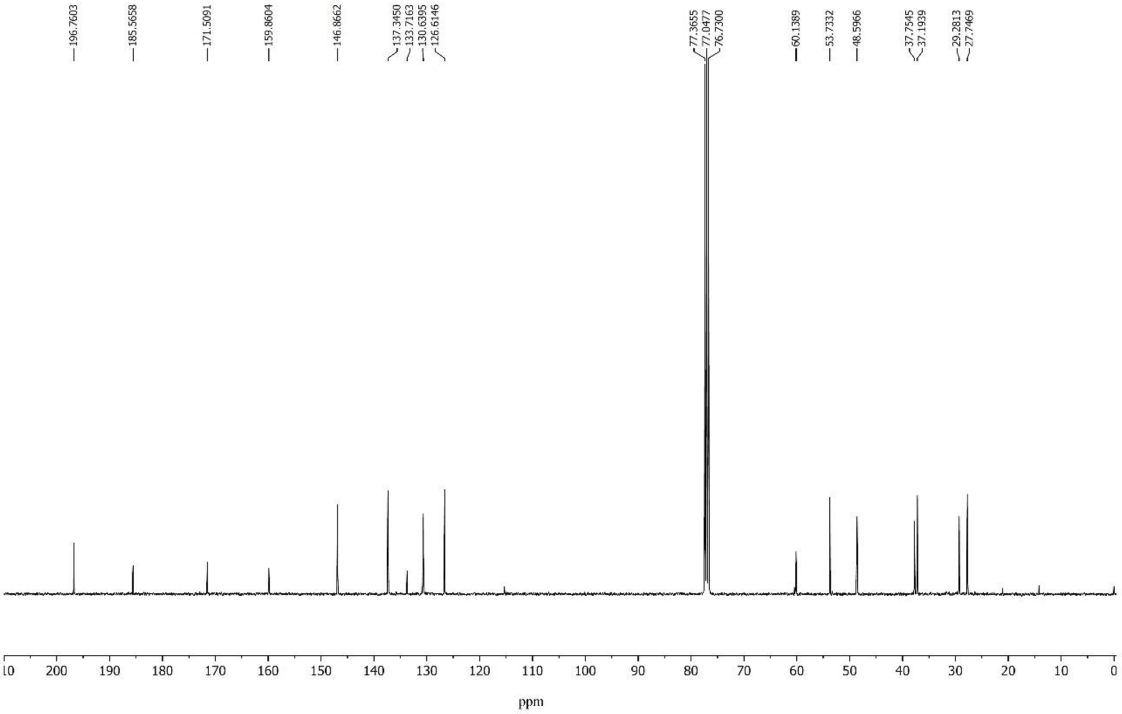

^{13}C NMR of compound **28**

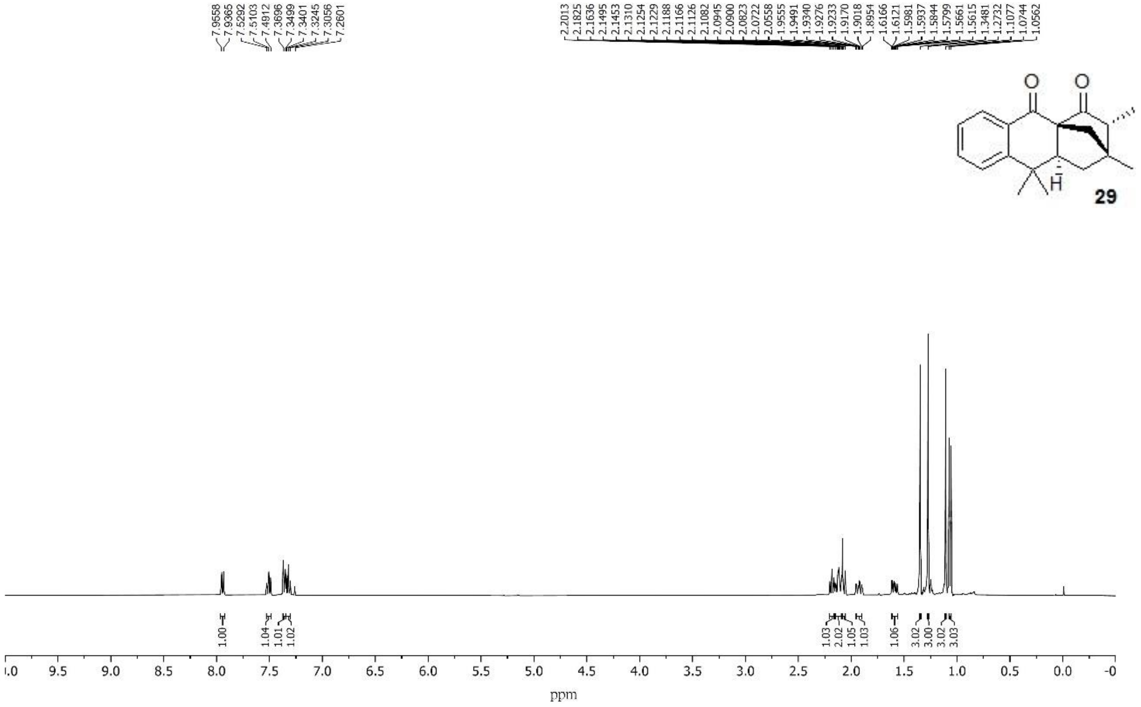

¹H NMR of compound **29**

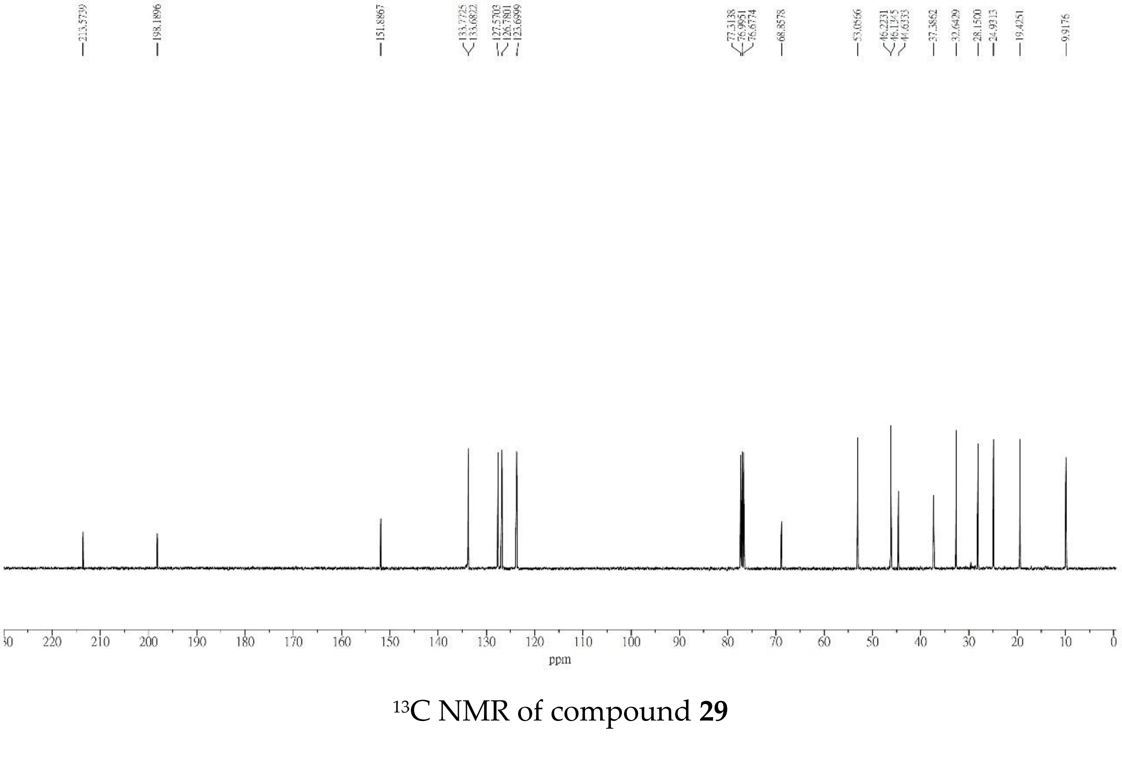

¹³C NMR of compound **29**

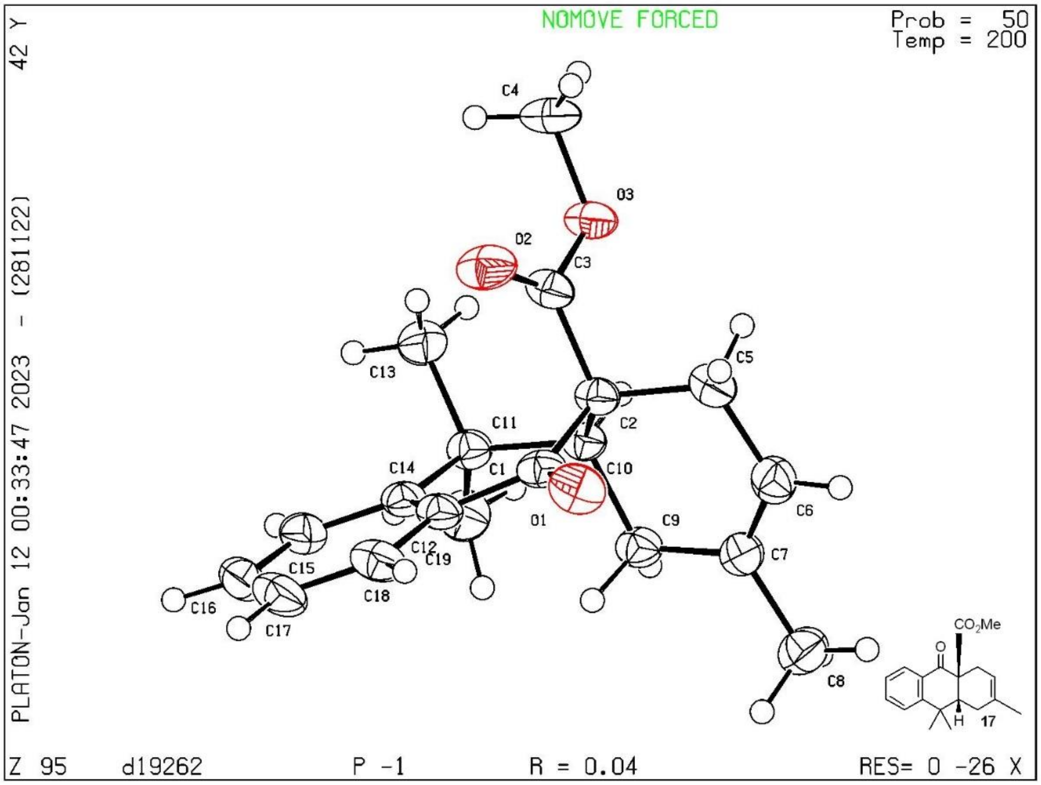

X-ray crystallographic structure of compound **17**

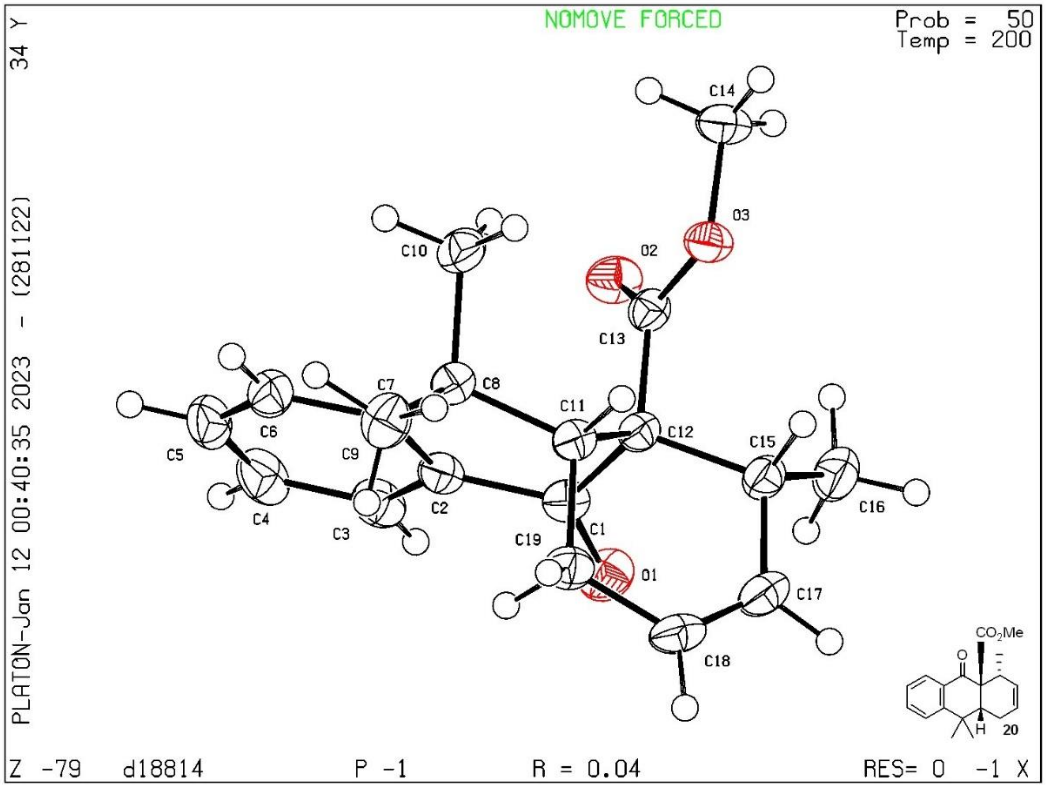

X-ray crystallographic structure of compound **20**

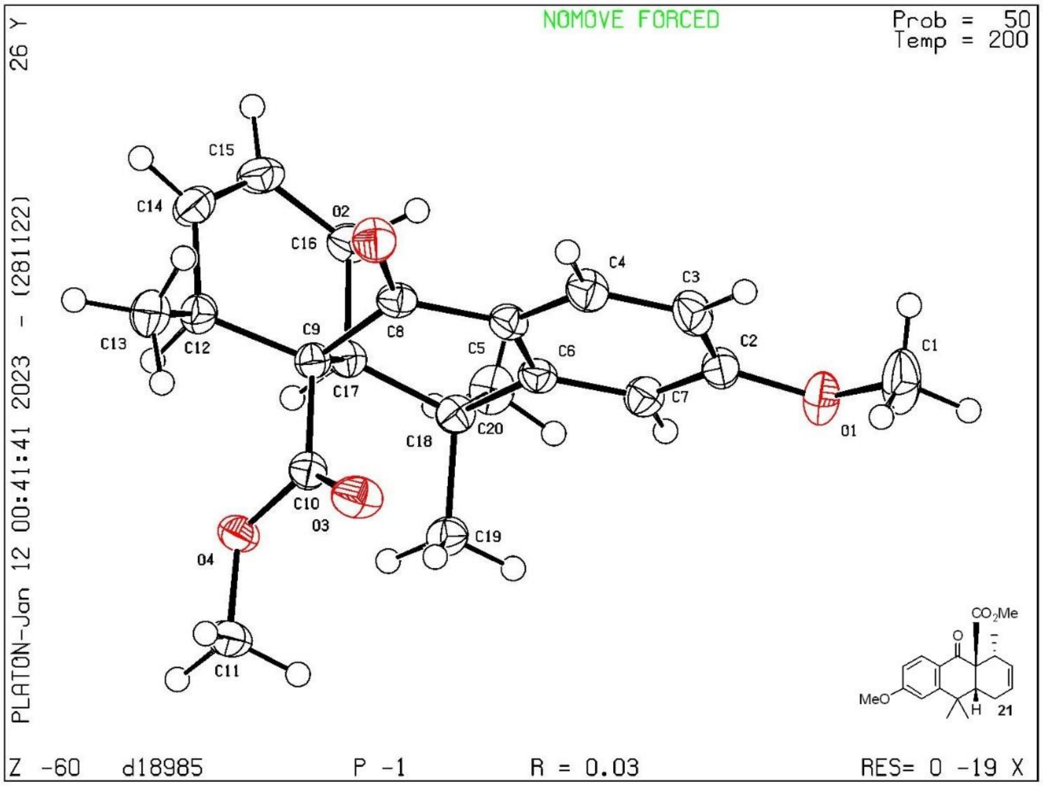

X-ray crystallographic structure of compound **21**

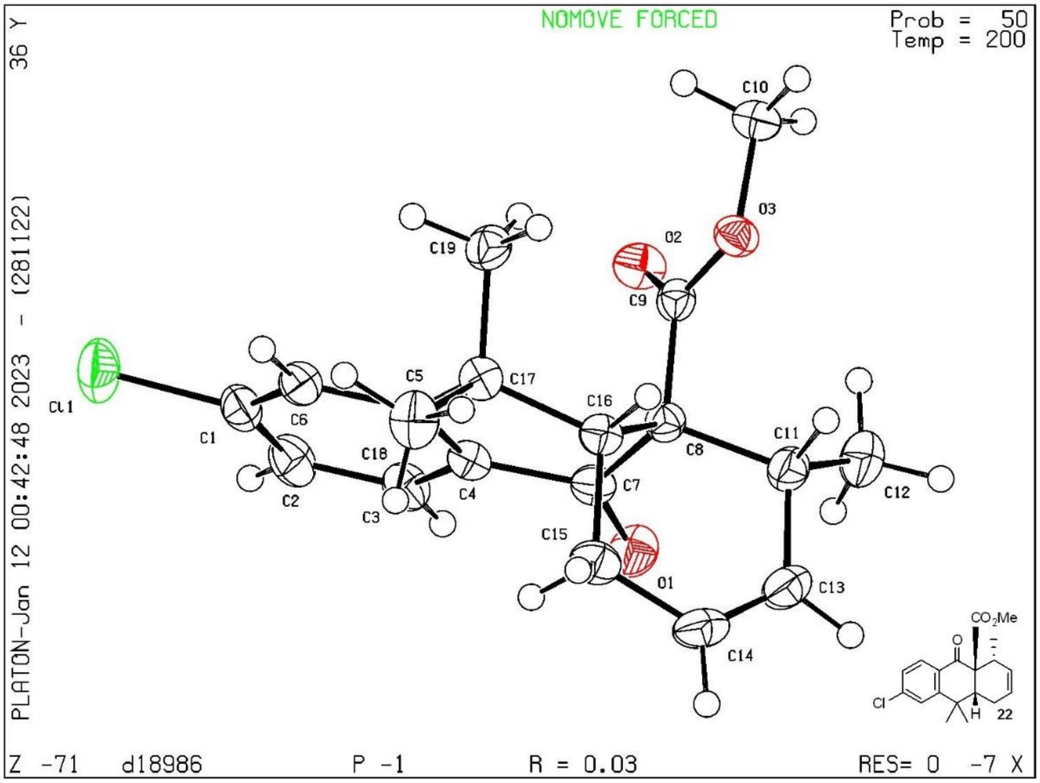

X-ray crystallographic structure of compound **22**

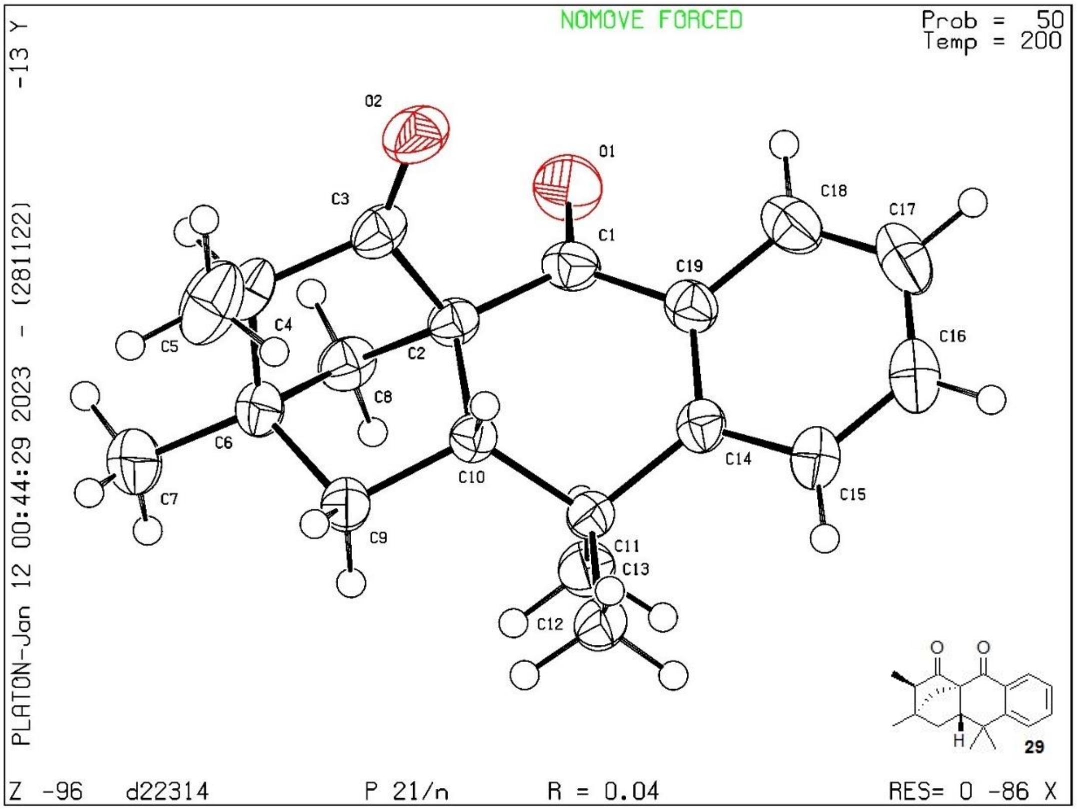

X-ray crystallographic structure of compound **29**

Table S1. Screening of Diels-Alder cycloaddition conditions.[a]

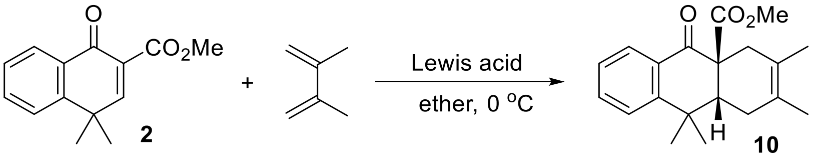

Entry	Diene (equiv.)	Lewis acid	Lewis acid (equiv.)	Time (h)	Yield (%)[b]
1	20	$SnCl_4$	2.5	3	84
2	20	$BF_3(OEt)_2$	2.5	3	30
3	20	$ZnCl_2$	2.5	3	72
4	20	$TiCl_4$	2.5	3	25
5	20	ZnI_2	2.5	3	74
6	20	BCl_3	2.5	3	63

[a]Reactions were performed using dienophile **2**, 2,3-dimethyl-1,3-butadiene and Lewis acid in ether (0.2M) at 0 °C under N_2 atmosphere. [b]Isolated yields.
